# Static strengths of circular hollow section stub column strengthened with carbon fiber reinforced polymer

**DOI:** 10.1371/journal.pone.0328047

**Published:** 2025-08-01

**Authors:** Chen Wei, Yang Yang

**Affiliations:** 1 Department of Civil Engineering, Sichuan College of Architectural Technology, Deyang, China; 2 Department of Mechanical and Information Engineering, Sichuan College of Architectural Technology, Deyang, China; Universiti Teknologi Malaysia, MALAYSIA

## Abstract

In previous studies, the strengthening effectiveness and feasibility of carbon fiber reinforced polymer (CFRP) were mainly evaluated through experimental research or numerical analysis. Although these methods can accurately provide estimates, both experimental and numerical assessment processes are costly in terms of time and cost. There is a need for reliable calculation formulae that can predict the static strengths of circular hollow section (CHS) stub columns in a more convenient and cost-saving manner. Therefore, this paper mainly conducted theoretical analysis and derived the calculation formulae. Furthermore, finite element (FE) analysis of CHS stub columns strengthened with CFRP was also conducted, and FE simulations were obtained. The accuracy of these formulae was validated by comparing the theoretical predictions with both FE simulations conducted by the authors and experimental results derived from previous studies. The maximum error was found to be no more than 10%. The cost-effective number of CFRP laminates bonded on the steel tube surface can be determined using the CFRP confinement coefficient *λ*, where *λ* balances the construction cost and the strengthening efficiency. A parametric study was also conducted to investigate the impact of steel strength, the number of CFRP laminates, and the bonding configuration of CFRP on the strengthening efficiency.

## 1. Introduction

Circular Hollow Section (CHS) steel tubes are crucial components in spatial truss structures, such as offshore jacket platforms and offshore wind turbines. However, the performance of these steel tubes is prone to deterioration due to corrosion and fatigue. It is necessary to strengthen or rehabilitate the steel tubes after a long-term service. Carbon fiber reinforced polymer (CFRP) is characterized by its high strength, corrosion and fatigue resistance, making it suitable for strengthening CHS steel tubes. Although the price of CFRP is relatively high, the convenient construction process helps save labor and time costs. Therefore, CFRP still has great application potential in strengthening steel tube components such as stub columns, concrete-filled steel tubes (CFST), and steel tubular-joints.

The effectiveness of CFRP strengthening for CHS stub columns has been confirmed by several researchers. Teng [1] and Haedir [2] experimentally proved that the strength of CHS stub columns strengthened with CFRP has been significantly improved. Furthermore, they also pointed out that the strengthening effect of CFRP is achieved by suppressing the buckling behavior of CHS stub columns, and this effect becomes stronger with the increase in the number of CFRP laminates. Additionally, Nabati [3] found that the CFRP strengthening technique can effectively restore the structural performance of deteriorated CHS stub columns, which results in a 75% recovery of the load-carrying capacity. Feng [4] pointed out that the CFRP strengthening technology is suitable for steel structures as well as other metal structures, such as aluminum structures. Moreover, other types of fiber reinforced polymers (FRPs), such as aramid fiber, can also improve the structural performance of CHS stub columns [5].

Similar to the hollow section steel tube stub columns, CFRP-strengthening can also improve the mechanical properties of concrete-filled steel tube (CFST) stub columns. Xiong [6] designed an experimental program, aiming to assess the strengthening efficiency of CFRP on circular CFST under preloading. Through the experimental research, Xiong [6] conducted an evaluation of the CFRP-strengthening effect on circular CFST and found that, after CFRP strengthening, a significant improvement in static strength was observed. Other researchers [7–9] also proved the effectiveness of CFRP-strengthening on CFST stub columns through experimental investigation.

CFRP can not only significantly improve the mechanical properties of single members such as steel tubular columns or concrete-filled steel tubular columns, but also have a positive enhancing effect on the performance of tubular joints. Sadat Hosseini [10] conducted FE analysis and found that the stress concentration factors (SCFs) of circular hollow section KT-shaped multiplanar (CHS-KT) joints are effectively reduced after CFRP strengthening, and a series of formulae to predict the SCF of CHS-KT joints are proposed. Similarly, the SCFs of circular hollow section T/Y-shaped planar (CHS-T/Y), circular hollow section X-shaped planar (CHS-X) and circular hollow section K-shaped planar (CHS-K) joints are all reduced after CFRP strengthening [11–16]. Xu [17] experimentally investigated the axial compressive behavior of circular hollow section TT-shaped multiplanar (CHS-TT) joints and found that the SCFs decreased by 14% and the fatigue life increased by 47%. Besides improving the fatigue strength, CFRP strengthening can also significantly improve the static strength of tubular joints. Mohamed [18] experimentally studied the corroded CHS-TT joints strengthened with CFRP and demonstrated that both the static strength and stiffness were enhanced.

Previous research findings have greatly demonstrated the effectiveness of CFRP-strengthening and have greatly promoted the application of CFRP in the field of structural reinforcement. CFRP enhances structural performance by providing effective confinement. Abbas et al. [19–22] demonstrated that CFRP provides non-negligible confinement effect among various confinement systems. In their studies, wrapping circular columns with 1-ply and 2-ply CFRP increased the confinement efficiency by approximately 134% and 183%, respectively, compared to unconfined columns. However, a series of specifications [23–26] indicate that once the number of CFRP laminates exceeds a certain threshold, adding more CFRP laminates will no longer contribute to improving the structural performance and will only increase the construction cost. Therefore, it is imperative to accurately assess the strengthening efficiency of CFRP. Researchers such as Xiong [6] and Wei [27] assessed the structural performance mainly through experimental research. Others, such as Tao [28], mainly evaluated the mechanical properties through numerical investigation. Although experimental or numerical analysis can accurately conclude the strengthening efficiency, both experimental and numerical assessment processes are costly in terms of time and resources. Different from the previous investigations, the authors in this study conducted a theoretical investigation and developed a series of formulae that provide a means to evaluate the static strengths of CHS stub columns strengthened with CFRP much more economically and conveniently. Ultimately, a theoretical evaluation method that balances strengthening efficiency and construction cost was developed based on the previous study.

## 2. Theoretical analysis

**Fig 1:**
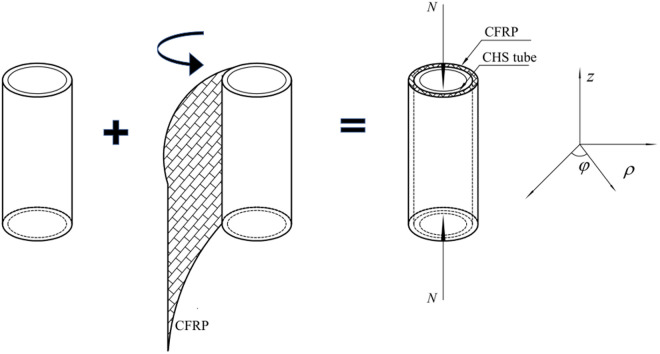
CFRP-strengthening construction process for CHS stub.

The CFRP-strengthening construction process for CHS stub column is shown in Fig 1. Compared to the welding and riveting strengthening techniques, the CFRP-strengthening process is much more convenient, as the strengthening materials are simply connected with the fundamental steel structures through bonding rather than welding or riveting.

**Fig 2:**
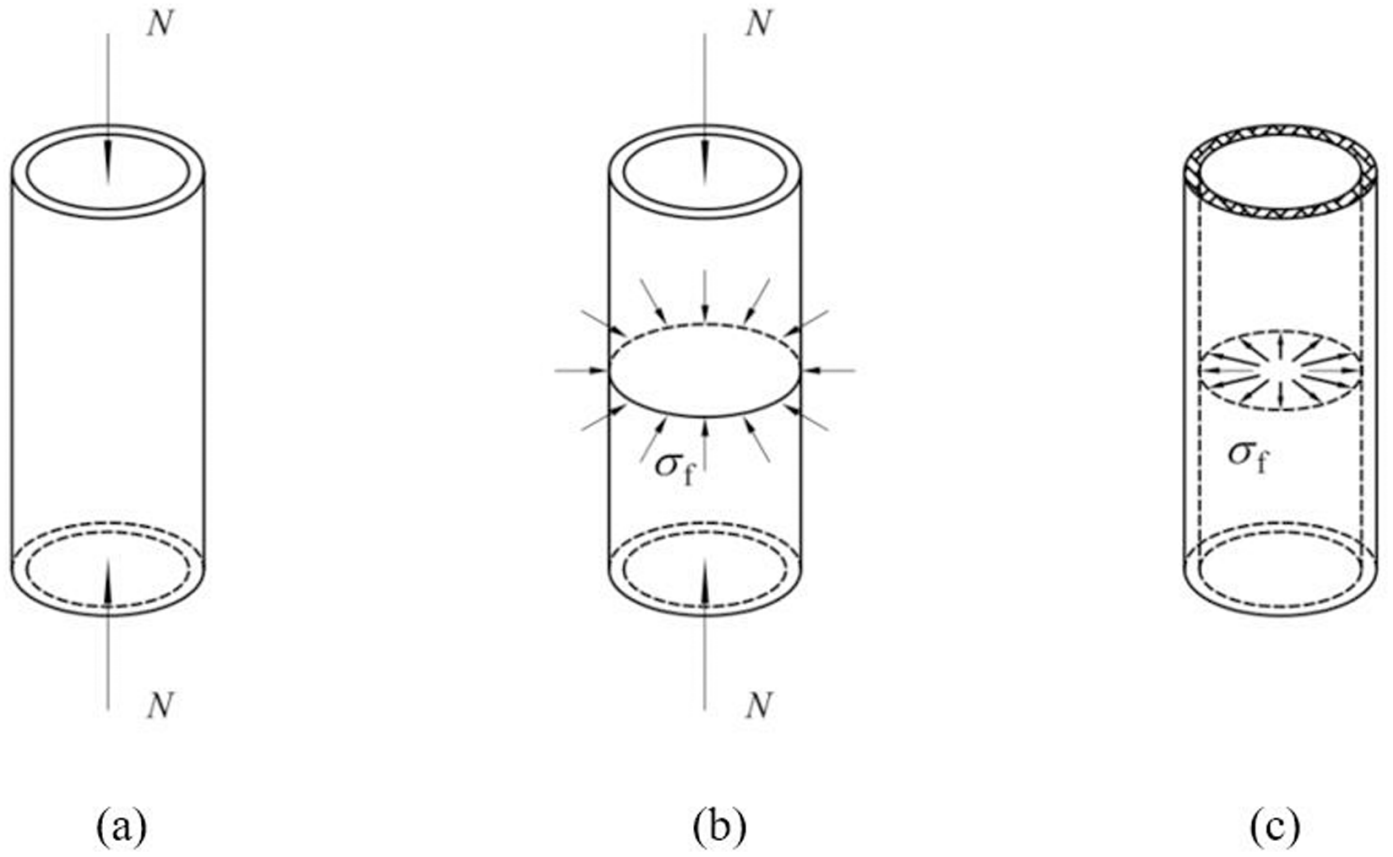
Analytical models of CFRP-strengthened CHS stub: (a) Steel tube; (b) Steel tube with CFRP; (c) CFRP.

The analytical models for CFRP-strengthened axially compressed CHS stub are illustrated in Fig 2. Here, *N* is the axial compressive load, while *σ*_f_ denotes the radial constraint stress imposed by the CFRP. After CFRP strengthening, the state of CHS steel tube changes from unidirectional compression, as depicted in Fig 2, to multidirectional compression, as shown in Fig 2.

### 2.1. Primary assumption

Based on the following primary assumptions, a theoretical analysis is conducted on CHS stubs strengthened with CFRP under axial compression.

**Fig 3:**
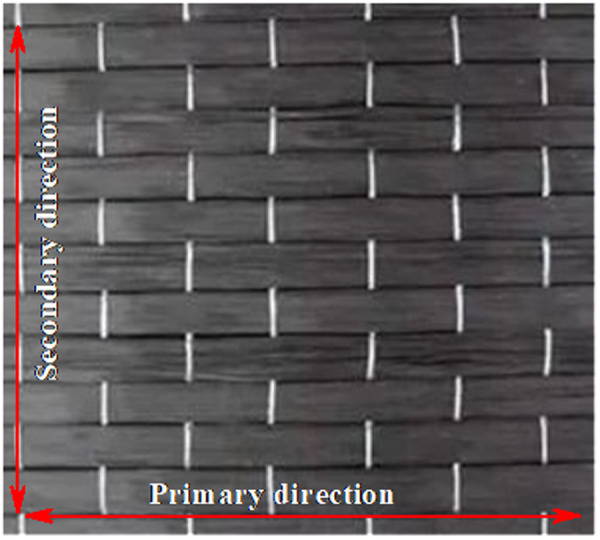
Direction definition of CFRP.

(1) During the axial loading process, the CFRP and the CHS stub deform in coordination, with no relative sliding occurring between them.
(2) The CHS stub, made of steel that satisfies the Von Mises yield criterion, exhibits ideal elastic-plastic behavior. In contrast, the CFRP behaves ideally as a linear-elastic material but fails immediately upon reaching its ultimate tensile strain.
(3) The CHS stub fails due to yielding under axial compression.
(4) The CFRP primarily exhibits stiffness in its fiber direction, i.e., primary direction. The stiffness of the resin matrix, contributes to defining the secondary direction stiffness of the CFRP, as shown in Fig 3.

### 2.2. Yield bearing capacity

**Fig 4:**
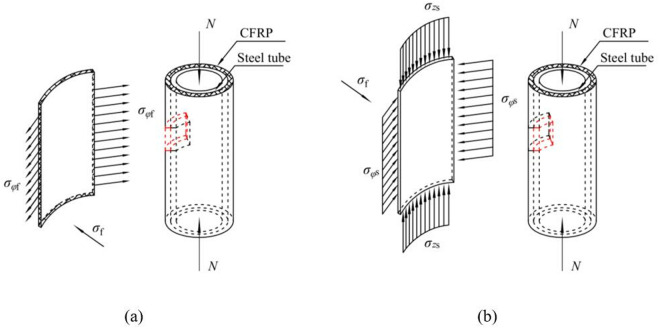
Stress components of CHS stub strengthened with CFRP: (a) CFRP; (b) Steel tube.


The stress components of CFRP and steel tube are shown in Fig 4. The differential equations of equilibrium (Eq. (1)), the geometrical equations (Eq. (2)), and the physical equations (Eq. (3)) for a CFRP-strengthened CHS stub column are presented as follows:

$$\left\{ \begin{array}{c} \frac{\partial \sigma_{\rho s}}{\partial \rho }+\frac{\sigma_{\rho s}-\sigma_{\varphi s}}{\rho }=0\\ \frac{\partial \sigma_{zs}}{\partial z}=0 \end{array}\right.$$ (1)

$$\left\{ \begin{array}{c} \varepsilon_{\rho s}=\frac{\partial u_{\rho }}{\partial \rho }\\ \varepsilon_{\varphi s}=\frac{u_{\rho }}{\rho } \end{array}\right.$$ (2)

$$\left\{ \begin{array}{c} \varepsilon_{\rho s}=\frac{1}{E_{s}}\left[\sigma_{\rho s}-\mu_{s}\left(\sigma_{\varphi s}+\sigma_{zs}\right)\right]\\ \varepsilon_{\varphi s}=\frac{1}{E_{s}}\left[\sigma_{\varphi s}-\mu_{s}\left(\sigma_{\rho s}+\sigma_{zs}\right)\right] \end{array}\right.$$ (3)

where *σ*_*ρ*s_, *σ*_*φ*s_ and *σ*_*z*s_ are the radial stress, hoop stress and axial stress of steel tube, *u*_*ρ*_ is the radial displacement, *ε*_*ρ*s_ and *ε*_*φ*s_ are the radial strain and hoop strain, respectively; *µ*_s_ and *E*_s_ are Poisson’s ratio, Young’s modulus of the CHS steel tube, respectively.

By substituting Eq. (2) into Eq. (3), the stress components (*σ*_*ρ*s_, *σ*_*φ*s_) can be expressed in terms of the radial displacement (*u*_*ρ*_):


$$\sigma_{\rho s}=\frac{{E\mathrm{\backslash nolimits}}_{s}}{1-{\mu_{s}}^{2}}\frac{\partial {u\mathrm{\backslash nolimits}}_{\rho }}{\partial \rho }+\frac{{E\mathrm{\backslash nolimits}}_{s}\mu_{s}}{1-{\mu_{s}}^{2}}\frac{{u\mathrm{\backslash nolimits}}_{\rho }}{\rho }+\frac{\mu_{s}}{1-\mu_{s}}\sigma_{z\mathrm{\backslash nolimits}s}$$ (4)

$$\sigma_{\varphi s}=\frac{{E\mathrm{\backslash nolimits}}_{s}}{1-{\mu_{s}}^{2}}\frac{{u\mathrm{\backslash nolimits}}_{\rho }}{\rho }+\frac{{E\mathrm{\backslash nolimits}}_{s}\mu_{s}}{1-{\mu_{s}}^{2}}\frac{\partial {u\mathrm{\backslash nolimits}}_{\rho }}{\partial \rho }+\frac{\mu_{s}}{1-\mu_{s}}\sigma_{z\mathrm{\backslash nolimits}s}$$ (5)

By substituting Eqs. (4) and (5) into Eq. (2), the radial displacement (*u*_*ρ*_) can be calculated and shown as follow:


$$u_{\rho }=C_{1}\rho +C_{2}\frac{1}{\rho }$$ (6)

where *C*_1_ and *C*_2_ are the undetermined constants.

By substituting Eq. (6) into Eqs. (5) and (4), the radial stress (*σ*_*ρ*s_) and hoop stress (*σ*_*φ*s_) can be expressed in terms of *C*_1_ and *C*_2_:


$$\sigma_{\rho s}=\frac{{E\mathrm{\backslash nolimits}}_{s}{C\mathrm{\backslash nolimits}}_{1}}{1-\mu_{s}}-\frac{{E\mathrm{\backslash nolimits}}_{s}{C\mathrm{\backslash nolimits}}_{2}}{\left(1+\mu_{s}\right)\rho^{2}}+\frac{\mu_{s}}{1-\mu_{s}}\sigma_{z\mathrm{\backslash nolimits}s}$$ (7)

$$\sigma_{\varphi s}=\frac{{E\mathrm{\backslash nolimits}}_{s}{C\mathrm{\backslash nolimits}}_{1}}{1-\mu_{s}}+\frac{{E\mathrm{\backslash nolimits}}_{s}{C\mathrm{\backslash nolimits}}_{2}}{\left(1+\mu_{s}\right)\rho^{2}}+\frac{\mu_{s}}{1-\mu_{s}}\sigma_{z\mathrm{\backslash nolimits}s}$$ (8)

The stress boundary conditions of the CHS stub column after CFRP strengthening are shown as follows:


$$\left\{ \begin{array}{c} \sigma_{\rho s\mathrm{\backslash nolimits}}=0\left(\rho =r\right)\\ \sigma_{\rho s\mathrm{\backslash nolimits}}=-\sigma_{f\mathrm{\backslash nolimits}}\left(\rho =R\right) \end{array}\right.$$ (9)

where *r* and *R* are the inner and the outer radius of the CHS steel tube, respectively; *σ*_f_ is the radial constraint stress imposed by CFRP.

By substituting Eq. (9) into Eq. (7), the undetermined constants *C*_1_ and *C*_2_ can be calculated as follows:


$$\left\{ \begin{array}{c} C_{1}=\frac{R^{2}\left(1-\mu_{s\mathrm{\backslash nolimits}}\right)}{\left(r^{2}-R^{2}\right)E_{s}}\sigma_{f\mathrm{\backslash nolimits}}-\frac{\mu_{s\mathrm{\backslash nolimits}}}{E_{s}}\sigma_{zs}\\ C_{2}=\frac{\left(1+\mu_{s}\right)r^{2}R^{2}}{E_{s}\left(r^{2}-R^{2}\right)}\sigma_{f\mathrm{\backslash nolimits}} \end{array}\right.$$ (10)

By substituting Eq. (10) into Eq. (7) and (8), making *ρ* = *R*, the stress components of steel tube can be expressed as follows:


$$\left\{ \begin{array}{c} \sigma_{\rho s\mathrm{\backslash nolimits}}=-\sigma_{f}\\ \sigma_{\varphi s\mathrm{\backslash nolimits}}=\frac{R^{2}+r^{2}}{r^{2}-R^{2}}\sigma_{f\mathrm{\backslash nolimits}}\\ \sigma_{zs\mathrm{\backslash nolimits}}=-\frac{N}{A_{s}+A_{c}} \end{array}\right.$$ (11)

where *N* is the axial compressive load; *A*_c_ is the cross-sectional area of CFRP jacket; *A*_s_ is cross-sectional area of steel tube.

In Eq. (11), both the radial stress (*σ*_*ρ*s_) and the hoop stress (*σ*_*φ*s_) are expressed in terms of the radial constraint stress (*σ*_f_). If the relationship between the radial constraint stress (*σ*_f_) and the axial stress (*σ*_*z*s_) is well-defined, then both the radial stress (*σ*_*ρ*s_) and the hoop stress (*σ*_*φ*s_) can be formulated in terms of the axial stress (*σ*_*z*s_). This could facilitate the determination of the yield strength and the yield bearing capacity of CFRP-strengthened axially compressed CHS stubs.

By substituting Eq. (11) into Eq. (3), the hoop strain (*ε*_*φ*s_) of steel tube after CFRP-strengthening can be derived as follow:


$${\varepsilon_{\varphi }}_{s}=\frac{R^{2}\left(1-\mu_{s\mathrm{\backslash nolimits}}\right)}{\left(r^{2}-R^{2}\right)E_{s}}\sigma_{f\mathrm{\backslash nolimits}}-\frac{\mu_{s}}{E_{s}}\sigma_{zs}+\frac{\left(1+\mu_{s\mathrm{\backslash nolimits}}\right)r^{2}}{E_{s}\left(r^{2}-R^{2}\right)}\sigma_{f\mathrm{\backslash nolimits}}$$ (12)

**Fig 5:**
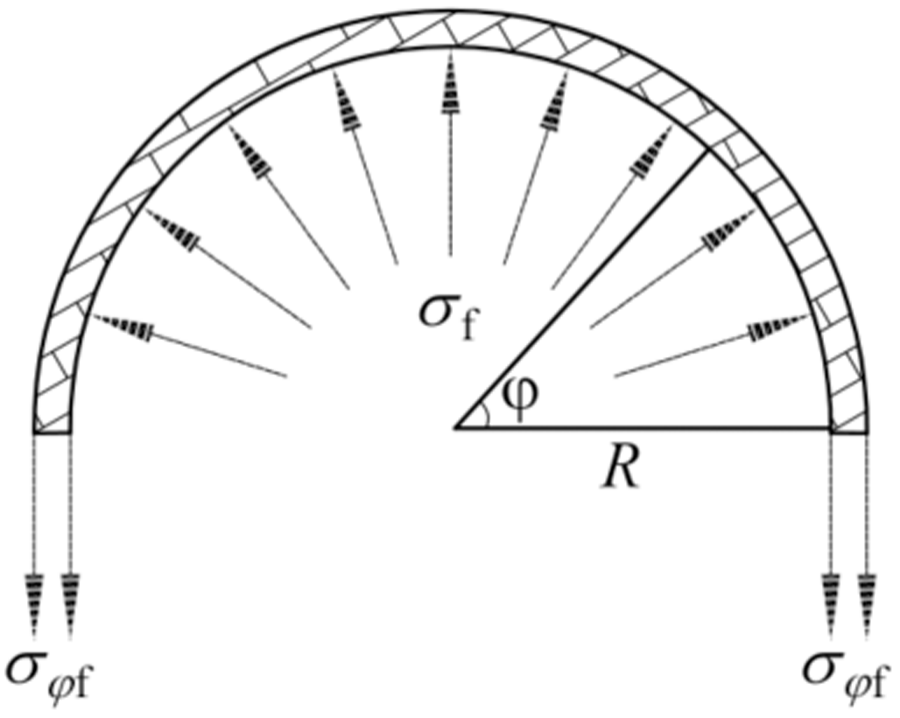
Sectional stress distribution of CFRP.


As shown in Fig 5, the sectional stress components of CFRP satisfy the below relations:

$$\varepsilon_{\varphi f}=\frac{\sigma_{f}R}{E_{ct}nt_{CFRP}}$$ (13)

where *E*_ct_ is the tensile modulus of CFRP; *t*_CFRP_ is the thickness of single CFRP laminate; *n* is the number of CFRP laminate.

During the axial loading process, the CFRP and the CHS stub undergo coordinated deformation, i.e., *ε*_*φ*f_ = *ε*_*φ*s_. By substituting Eq. (13) into Eq. (12), the relationship between radial constraint stress *σ*_f_ and axial stress *σ*_*z*s_ can be defined as follows:


$$\sigma_{f\mathrm{\backslash nolimits}}=\eta \left(r^{2}-R^{2}\right)\sigma_{zs}$$ (14)

$$\eta =\frac{\alpha \mu_{s}nt_{CFRP}}{nt_{CFRP}\alpha \left(R^{2}+r^{2}\right)+\left(r^{2}-R^{2}\right)\left(nt_{CFRP}\alpha \mu_{s}-R\right)}$$ (15)

where *α* is the modulus ratio of CFRP to CHS stub.

By combining Eq. (14) and Eq. (11), the radial stress (*σ*_*ρ*s_) and the hoop stress (*σ*_*φ*s_) can be formulated in terms of the axial stress (*σ*_*z*s_), and all relevant stress components are re-expressed as follows:


$$\left\{ \begin{array}{c} \sigma_{\rho s\mathrm{\backslash nolimits}}=-\eta \left(r^{2}-R^{2}\right)\sigma_{zs}\\ \sigma_{\varphi s\mathrm{\backslash nolimits}}=\eta \left(r^{2}+R^{2}\right)\sigma_{zs}\\ \sigma_{zs\mathrm{\backslash nolimits}}=-\frac{N}{A_{s}+A_{c}} \end{array}\right.$$ (16)

The stress components of steel tube satisfy the Von Mises yield criterion:


$$f_{y\mathrm{\backslash nolimits}}=\sqrt{\frac{1}{2}\left[{\left(\sigma_{\rho s\mathrm{\backslash nolimits}}-\sigma_{\varphi s\mathrm{\backslash nolimits}}\right)}^{2}+{\left(\sigma_{\varphi s\mathrm{\backslash nolimits}}-\sigma_{zs\mathrm{\backslash nolimits}}\right)}^{2}+{\left(\sigma_{zs\mathrm{\backslash nolimits}}-\sigma_{\rho s\mathrm{\backslash nolimits}}\right)}^{2}\right]}$$ (17)

where *f*_y_ is the yield strength of steel tube.

By substituting Eq. (16) into Eq. (17), the yield strength (*σ*_*z*sy_) and yield bearing capacity (*N*_y_) of the CHS stub column are determined as follows:


$$\left|\sigma_{zsy}\right|=\frac{f_{y\mathrm{\backslash nolimits}}}{\sqrt{\eta^{2}\left(R^{4}+3r^{4}\right)-2\eta R^{2}+1}}$$ (18)

$$N_{y}=\left|\sigma_{zsy}\right|\cdot (A_{s}+A_{c})$$ (19)

When CFRP is placed along hoop direction, Eq. (14) needs to be modified as follows:


$$\left\{ \begin{array}{c} \alpha =\alpha_{c}\\ n=n_{H} \end{array}\right.$$ (20)

where *α*_c_ is the modulus ratio of CFRP to CHS steel tube, *n*_H_ is the number of CFRP laminates bonded along the hoop direction.

When CFRP is alternatively placed, i.e., placed along hoop and longitudinal direction, Eq. (14) also needs to be adjusted as follows:


$$\left\{ \begin{array}{c} \alpha =\alpha_{c}+\alpha_{m}\\ n=n_{H}+n_{L} \end{array}\right.$$ (21)

where *α*_m_ is the modulus ratio of resin matrix to CHS steel tube, *n*_L_ is the number of CFRP laminates bonded along the longitudinal direction.

### 2.3. Ultimate bearing capacity

After yielding of CFRP-strengthened CHS stub column, the strain of CFRP continues to increase. As the strain of CFRP increases, its restraining effect on CHS stub column is effectively enhanced, resulting in a continuous improvement in the load bearing capacity. The ultimate bearing capacity of the CFRP-strengthened CHS stub is attained when CFRP fractures, i.e., when the strain of CFRP (*ε*_*φ*f_) reaches the ultimate tensile strain (*ε*_f_).

By substituting the ultimate tensile strain (*ε*_f_) into Eq. (13), the ultimate radial constraint stress (*σ*_fu_) can be attained. Subsequently, by substituting the ultimate radial constraint stress (*σ*_fu_) into Eq. (11), both the ultimate hoop stress (*σ*_*ρ*su_) and the ultimate radial stress (*σ*_*φ*su_) can be determined. Finally, by substituting *σ*_*ρ*su_ and *σ*_*φ*su_ into Eq. (17), the ultimate axial stress *σ*_*z*su_ and the ultimate bearing capacity *N*_u_ are achieved as follows:


$$\left|\sigma_{zsu}\right|=\left(\sqrt{{f_{y\mathrm{\backslash nolimits}}}^{2}-3{\left[\frac{\left(r-t_{s}\right)f_{ct}d_{CFRP}}{t_{s}D}\right]}^{2}}+\frac{\left(r+t_{s}\right)f_{ct}d_{CFRP}}{t_{s}D}\right)$$ (22)

$$N_{u\mathrm{\backslash nolimits}}=\left|\sigma_{zsu}\right|(A_{s}+A_{c})$$ (23)

where *t*_s_ is the tube thickness, *D* is the outer diameter of steel tube, *f*_ct_ is the tensile strength of CFRP, *d*_CFRP_ is the total thickness of CFRP jackets.

When CFRP is placed along hoop direction:


$$d_{CFRP}=n_{H}t_{CFRP}$$ (24)

When CFRP is alternatively placed, i.e., placed along hoop and longitudinal direction:


$$d_{CFRP=}n_{H}\cdot t_{CFRP}+n_{L}\cdot t_{CFRP}\cdot \frac{E_{er}}{E_{ct}}$$ (25)

where *E*_er_ is the modules of resin matrix.

According to Eqs. (22) and (23), the following relation should be satisfied:


$${f_{ys}}^{2}-3{\left[\frac{\left(r-t_{s}\right)f_{ct}d_{CFRP}}{t_{s}D}\right]}^{2}\geq 0$$ (26)

Normally, the outer diameter (*D*) of CHS steel tube is significantly larger than its tube thickness (*t*_s_). Therefore, Eq. (26) can be simplified as follows:


$$\frac{f_{ct}d_{CFRP}}{f_{ys}t_{s}}\leq \frac{2\sqrt{3}}{3}$$ (27)

Given that the outer diameter *D* is much larger than the tube thickness *t*_s_, the outer and inner diameters (*D* and *d*, respectively) are assumed to be approximately equal. Therefore, Eq. (27) can be transformed as follows:


$$\lambda =\frac{f_{ct}d_{CFRP}}{f_{ys}t_{s}}=\frac{\pi Df_{ct}d_{CFRP}}{\pi df_{ys}t_{s}}=\frac{A_{c}f_{ct}}{A_{s}f_{ys}}\leq \frac{2\sqrt{3}}{3}=1.155$$ (28)

Increasing the number of CFRP laminates without upper limitation may not improve the structural performance of CHS stub column as designed. When CHS stub columns are excessively strengthened with CFRP laminates, the steel tube experiences significant constraint, leading to inward deformation. This inward deformation causes delamination between CFRP and steel tube, resulting in failure of the strengthened CHS stub column due to CFRP delamination rather than CFRP fracture. Consequently, the tensile strength of CFRP cannot be fully utilized, and the CFRP-strengthening efficiency does not increase with the addition of more CFRP layers. Once the number of CFRP laminates exceeds a certain threshold, further layers no longer contribute to improving the structural performance and only increase construction cost. The economically optimal number of CFRP laminates can be decided using the CFRP confinement constant *λ*. Importantly, CFRP will fail due to fracture if *λ* is less than 1.155, and in this situation, the performance of CHS stub column is proportional to the number of CFRP layers.

## 3. Finite element analysis

During the theoretical analysis, CFRP experiences instantaneous failure upon reaching the ultimate tensile strain, and the CHS steel tube assumes to be geometric perfect. However, in practice, the failure behavior of CFRP is gradually developed and the geometric perfection for a CHS steel tube is difficult to maintain. In general, theoretical analysis simplified the actual situation.

**Fig 6:**
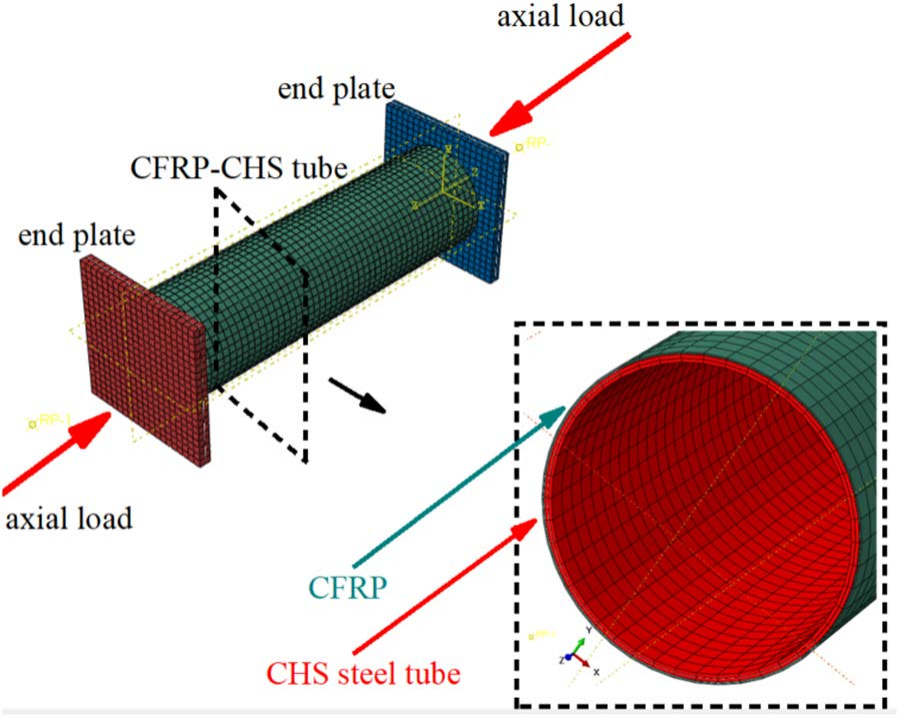
FE model of CFRP-strengthened CHS stub column.

To validate the accuracy of the theoretical formulae and obtain a proper conclusion, a finite element investigation of CFRP-strengthened CHS stub columns should be carried out. The FE model is shown in Fig 6.

### 3.1. Model details

Previous studies have conducted experimental studies on CHS stub columns with CFRP strengthening. The details of specimens are listed in Table 1, where *D*, *t* and *L* represent the tube diameter, tube thickness, and tube length, *E*_s_ is the Young’s module, *f*_ys_ is the steel yield strength. Regarding the configuration of CFRP, ‘L’ and ‘H’ represent the longitudinal (fiber parallel to the axis of steel tube) CFRP laminate and the hoop (fiber perpendicular to the axis of steel tube) CFRP laminate placed on the surface of the CHS stub columns, respectively. Therefore, ‘1H1L’ indicates that one hoop layer and one longitudinal layer of CFRP are sequentially placed, while ‘2H2L’ signifies that two hoop layers and two longitudinal layers of CFRP are alternately placed. Similarly, ‘1H’ (or ‘2H’, ‘3H’) means that one (or two, three) layer(s) of CFRP are placed along the hoop direction.

**Table 1:** Model details.

Ref.	ID	*D* mm	*t* mm	*L* mm	*D*/*t*	CFRP
**[** **27** **]**	4-1T1L-0	89.0	4.0	300.0	22.3	1H1L
	4-2T2L-0	89.0	4.0	300.0	22.3	2H2L
	2-2T-0	85.0	2.0	300.0	42.5	2H
**[** **2** **]**	CF-1A	87.2	2.3	279.0	37.9	1H1L
	CF-1B	87.2	2.3	279.0	37.9	2H2L
	CF-2A	86.4	1.9	276.5	45.5	1H1L
	CF-2B	86.4	2.0	276.5	43.2	2H2L
	CF-3A	85.7	1.6	274.2	53.6	1H1L
**[** **1** **]**	ST-F1	166.0	4.2	450.0	39.5	1H
	ST-F2	165.0	4.2	450.0	39.3	2H
	ST-F3	165.0	4.2	450.0	39.3	3H
**[** **29** **]**	S168L5T3C	168.0	5.0	500.0	33.6	3H
	S168L5T5C	168.0	5.0	500.0	33.6	3H
	S168L5T7C	168.0	5.0	500.0	33.6	3H
	S140L5T3C	140.0	5.0	500.0	28.0	3H
	S140L5T5C	140.0	5.0	500.0	28.0	3H
	S140L5T7C	140.0	5.0	500.0	28.0	3H
	S140L8T3C	140.0	5.0	800.0	28.0	3H
	S140L8T5C	140.0	5.0	800.0	28.0	3H
	S140L8T7C	140.0	5.0	800.0	28.0	3H
**[** **30** **]**	S-1A-C	168.0	5.0	500.0	33.6	3H3L
	S-2A-C	180.0	5.0	1500	36.0	3H3L
	S-2A-C1	180.0	5.0	1500	36.0	3H3L
**[** **5** **]**	S1-20-A1	58.0	1.0	200.0	58.0	1H
	S1-20-A2	58.0	1.0	200.0	58.0	2H
	S1-20-A3	58.0	1.0	200.0	58.0	3H
	S1.5-20-A1	58.0	1.5	200.0	38.7	1H
	S2-20-A1	58.0	2.0	200.0	29.0	1H
	S2-20-A2	58.0	2.0	200.0	29.0	2H
**[** **31** **]**	SCT50-0	49.7	3.1	150.0	16.0	–
	SCT50-1	49.7	3.1	150.0	16.0	1H1L
	SCT50-2	49.7	3.1	150.0	16.0	2H2L
	SCT50-3	49.7	3.1	150.0	16.0	3H3L

Note: ‘-’ means that specimen ‘SCT50-0’ has not been strengthened with CFRP.

### 3.2. Material property

The tensile coupon test for the CHS steel tube was conducted in the previous experimental investigation, and the material properties of the steel are listed in Table 2, where *f*_y_, *E*_s_, *f*_u_ and *μ*_s_ represent the yield strength, tensile modulus, ultimate strength, and Poisson’s ratio, respectively. The deformation of the end plates on both sides of the CHS stub column can be ignored, i.e., the Young’s modulus of the end plate is assumed to be very large, so that the end plates are treated as rigid body in the FE analysis.

**Table 2:** Material properties of CHS steel tube.

Ref.	ID	*E*_s_ GPa	*f*_y_ MPa	*f*_u_ MPa	*μ*_s_
**[** **27** **]**	4-1T1L-0	200.3	335.0	454.0	0.28
	4-2T2L-0	200.3	335.0	454.0	0.28
	2-2T-0	200.3	335.0	454.0	0.28
**[** **2** **]**	CF-1A	209.5	455.0	507.0	0.3
	CF-1B	209.5	455.0	507.0	0.3
	CF-2A	209.5	455.0	507.0	0.3
	CF-2B	209.5	455.0	507.0	0.3
	CF-3A	209.5	455.0	507.0	0.3
**[** **1** **]**	ST-F1	201.0	333.0	370.0	0.34
	ST-F2	201.0	333.0	370.0	0.34
	ST-F3	201.0	333.0	370.0	0.34
**[** **29** **]**	S168L5T3C	190.0	408.0	604.0	0.28
	S168L5T5C	195.0	428.0	611.0	0.28
	S168L5T7C	199.0	393.0	554.0	0.28
	S140L5T3C	207.0	442.0	612.0	0.28
	S140L5T5C	215.0	472.0	660.0	0.28
	S140L5T7C	207.0	414.0	581.0	0.28
	S140L8T3C	207.0	442.0	612.0	0.28
	S140L8T5C	215.0	472.0	660.0	0.28
	S140L8T7C	207.0	414.0	581.0	0.28
**[** **30** **]**	S-1A-C	200.0	345.5	499.0	0.3
	S-2A-C	200.0	345.5	499.0	0.3
	S-2A-C1	200.0	345.5	499.0	0.3
**[** **5** **]**	S1-20-A1	196.0	370.0	549.0	0.3
	S1-20-A2	196.0	370.0	549.0	0.3
	S1-20-A3	196.0	370.0	549.0	0.3
	S1.5-20-A1	208.0	272.0	410.0	0.3
	S2-20-A1	208.0	272.0	410.0	0.3
	S2-20-A2	208.0	272.0	410.0	0.3
**[** **31** **]**	SCT50-0	70.3	291.0	295.0	0.36
	SCT50-1	70.3	291.0	295.0	0.36
	SCT50-2	70.3	291.0	295.0	0.36
	SCT50-3	70.3	291.0	295.0	0.36

The CFRP is an ideal linear-elastic material before fracture failure. To properly define the material properties of CFRP in FE analysis is crucial, as it directly influences the accuracy of FE results. In this regard, Abbas et al. [19,22] have successively conducted similar analyses on the axial compressive behavior of reinforced-concrete columns. As suggested by Abbas et al. [19,22], the structural performance of CFRP can be properly defined by the ‘lamina’ option in ABAQUS. The failure process of CFRP gradually evolves, and the ‘Hashin Damage’ model is specifically used to explain the failure behavior of materials like CFRP in ABAQUS. The material properties of FRP are listed in Table 3, where *f*_ct_, *E*_ct_, *f*_cc_, *E*_cc_, *ε*_c_ and *t*_FRP_ represent the tensile strength, tensile modulus, compressive strength, compressive modulus, tensile strain and thickness of single FRP laminate, respectively.

**Table 3:** Mechanical properties and geometric details of FRP laminate.

Ref.	*f*_ct_ MPa	*E*_ct_ GPa	*ε*_c_	*f*_cc_ MPa	*E*_cc_ GPa	*t*_FRP_ mm
**[** **27** **]**	3720	228	0.016	92.6	3.03	0.167
**[** **1** **]**	1825.5	80.1	0.0228	N/A	N/A	0.170
**[** **2** **]**	1830	230	N/A	32	1.9	0.176
**[** **29** **]**	3720	228	0.016	N/A	N/A	0.167
**[** **30** **]**	2650	238	0.014	N/A	N/A	0.167
**[** **5** **]**	1400	79	N/A	132	5.5	0.250
**[** **31** **]**	2034	71.5	N/A	10.7	N/A	0.650

Note: ‘N/A’ means that relevant information is not provided by the previous research.

### 3.3. Boundary condition and mesh discretization

#### 3.3.1. Boundary condition.

During the experimental investigation, the upper part of the universal testing machine was completely fixed, while the lower part can only be axially moved to apply the axial compressive pressure to the specimens [27]. The boundary condition in the FE analysis should be consistent with the experimental setup, i.e., the top end of FE model is completely fixed and the bottom end can only be moved axially.

#### 3.3.2. Mesh discretization.

Considering the computational efficiency and accuracy, the shell element S4R is selected to discretize the geometry of the CHS stub column. Considering that CFRP exhibits significant stiffness in fiber direction, the membrane element M3D4R is chosen to discretize the geometry of CFRP, as it is well-suited for capturing in-plane behavior. According to the suggestion of Tao et al. [28], the global mesh size should be set to 1/40 of the length of the steel tube in numerical model, ensuring both computational efficiency and accuracy. As a result, the global mesh size here is determined to be 7 mm.

#### 3.3.3. Mesh sensitivity analysis.

Tao et al. [28] successfully conducted finite element analysis (FEA) on the mechanical behavior of CFST stub columns under axial compression, achieving a balance between computational efficiency and accuracy through optimized mesh discretization strategies. Building on this methodology, the present study adopted an analogous modeling approach to discretize the geometric configuration of CHS stub columns strengthened with CFRP and performed FEA simulations. However, due to inherent structural differences between the analyzed components, the direct application of Tao’s meshing strategy may introduce discrepancies in the simulated responses. To address this uncertainty, a mesh sensitivity analysis is required to evaluate the convergence of results, thereby ensuring the reliability of numerical predictions. Abbas et al. [19,22] have implemented effective meshing strategies to ensure the mesh convergence requirement in their research. To ensure the accuracy of our analysis, similar mesh size verification procedures are conducted in this section.

**Fig 7:**
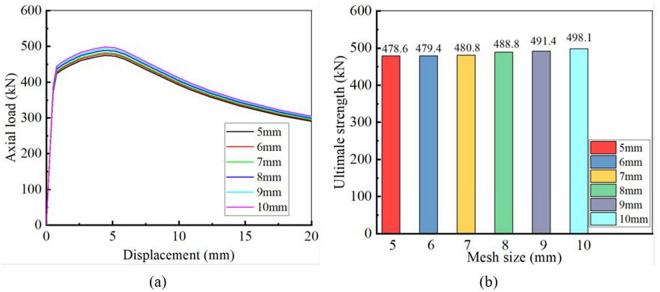
Comparison of FEA results for different mesh sizes: (a) Load-displacement curves; (b) Ultimate strengths.

According to Tao’s meshing strategy, the mesh size is set to 7 mm. For the CHS stub column “4-1T1L-0” listed in Table 1, geometric meshes with sizes of 5 mm, 6 mm, 7 mm, 8 mm, 9 mm, and 10 mm were created separately. Then, FEA were performed to obtain the load-displacement curves and the ultimate strengths for different mesh sizes. As illustrated in Fig 7, when the mesh sizes were 5 mm, 6 mm, and 7 mm, the load-displacement curves showed no significant differences, and the ultimate strengths stabilized at 480 kN. Discretizing the geometric part with a 7 mm mesh size can meet both efficiency and accuracy requirements. The meshing strategy introduced by Tao et al. [28] to discretize CFST stub columns can also be applied to mesh CHS stub columns similar to “4-1T1L-0” in this study.

### 3.4. Interaction

The contact relationship between CFRP and the CHS steel tube can be established in the “interaction” module of ABAQUS. During our previous experimental analysis in Ref. [27], we observed that CFRP deforms in coordination with the CHS tube, and no debonding occurs between them before the tube loses its load-bearing capacity. In finite element analysis, the “Tie” constraint is simply adopted to define the contact relationship between CFRP and the CHS tube. This approach is consistent with experimental observations and significantly reduces the analysis time cost. Similarly, a comparable approach for defining contact relationships has been adopted in studies by Xiong [6] and Huang [8].

### 3.5. Geometrical imperfections

The CHS stub is prone to buckling under axial compression, and geometric imperfection accelerates this buckling process. Therefore, the effect of geometric imperfection on the performance of CHS stub should be fully considered in the FE analysis. To introduce geometric imperfection into the FE models, eigenvalue buckling analysis and nonlinear buckling analysis are carried out successively. The first buckling mode is obtained through eigenvalue buckling analysis. The shape of this first buckling mode is then used as the initial geometric imperfection in nonlinear buckling analysis, with an imperfection amplitude of 2% [27].

## 4. Verification

**Fig 8:**
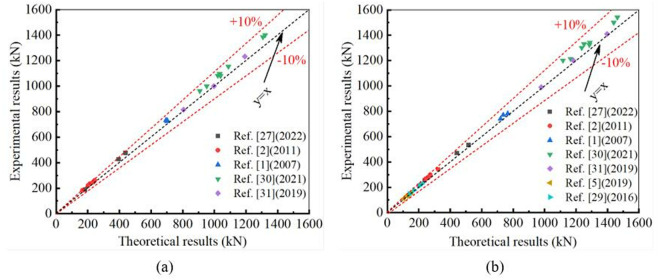
Comparison between experimental results and theoretical predictions: (a) Yield strengths; (b) Ultimate strengths.

The theoretical yield strength *N*_y_ and ultimate strength *N*_u_ were obtained based on Eqs. (19) and (23), respectively. Through experimental investigation, many researchers obtained the experimental results of the specimen, which include the yield strength (*N*_EX,y_) and the ultimate strength (*N*_EX,u_). When comparing the theoretical results with the experimental investigations, the average (AVR) and standard deviation (S.D.) of *N*_y_/*N*_EX,y_ are 0.94 and 0.02; the average and standard deviation of *N*_u_/*N*_EX,u_ are 0.95 and 0.02. Details can be found in Table 4. The maximum error between theoretical predictions and experimental results is less than 10%, as shown in Fig 8. Theoretical predictions are in good agreement with the experimental results, and theoretical formulae can provide proper evaluations of the structural performance of CHS stub columns strengthened with CFRP.

**Table 4:** Comparisons of load bearing capacity.

Ref.	ID	*N*_y_ /kN	*N*_u_ /kN	*N*_EX,y_ /kN	*N*_EX,u_ /kN	*N*_FE,y_ /kN	*N*_FE,u_ /kN	$\frac{N_{y}}{N_{EX,y}}$	$\frac{N_{u}}{N_{EX,u}}$	$\frac{N_{y}}{N_{FE,y}}$	$\frac{N_{u}}{N_{FE,u}}$
[27]	4-1T1L-0	394	442	429	472	430	477	0.92	0.94	0.92	0.93
	4-2T2L-0	437	516	479	535	485	543	0.91	0.96	0.91	0.95
	2-2T-0	182	236	190	256	193	260	0.96	0.92	0.94	0.91
[2]	CF-1A	235	273	248	299	255	293	0.95	0.91	0.92	0.93
	CF-1B	244	324	261	341	262	343	0.93	0.95	0.93	0.94
	CF-2A	203	244	223	267	222	269	0.91	0.91	0.91	0.91
	CF-2B	215	263	236	281	231	277	0.91	0.94	0.93	0.95
	CF-3A	167	201	183	214	177	213	0.91	0.94	0.94	0.94
[1]	ST-F1	701	720	724	740	729	757	0.97	0.97	0.96	0.95
	ST-F2	703	737	731	771	737	783	0.96	0.96	0.95	0.94
	ST-F3	695	766	733	782	740	794	0.95	0.98	0.94	0.96
[29]	S168L5TC	1307	1463	1385	1541	1322	1529	0.94	0.95	0.99	0.96
	S168L5TC	1321	1440	1401	1500	1385	1531	0.94	0.96	0.95	0.94
	S168L5TC	1089	1285	1154	1324	1103	1389	0.94	0.97	0.99	0.93
	S140L5TC	1034	1251	1096	1330	1121	1385	0.94	0.94	0.92	0.90
	S140L5TC	1033	1251	1097	1331	1118	1368	0.94	0.94	0.92	0.91
	S140L5TC	952	1165	1000	1214	1003	1238	0.95	0.96	0.95	0.94
	S140L8TC	1039	1288	1081	1342	1095	1396	0.96	0.96	0.95	0.92
	S140L8TC	1022	1236	1083	1301	1092	1353	0.94	0.95	0.94	0.91
	S140L8TC	908	1117	963	1201	985	1237	0.94	0.93	0.92	0.90
[30]	S-1A-C	1193	1398	1232	1410	1215	1402	0.97	0.99	0.98	0.99
	S-2A-C	805	979	816	990	808	995	0.99	0.99	0.99	0.98
	S-2A-C1	997	1183	1000	1200	989	1195	0.99	0.99	1.00	0.99
[5]	S1-20-A1	90	105	–	110	98	115	N/A	0.95	0.92	0.91
	S1-20-A2	108	123	–	132	110	131	N/A	0.93	0.98	0.94
	S1-20-A3	120	136	–	145	127	149	N/A	0.94	0.94	0.91
	S1.5-20A1	116	129	–	135	121	142	N/A	0.96	0.96	0.91
	S2-20-A1	119	144	–	154	133	157	N/A	0.94	0.90	0.92
	S2-20-A2	136	153	–	161	132	155	N/A	0.95	1.03	0.99
[31]	SCT50-0	128	141	–	148	137	150	N/A	0.95	0.93	0.94
	SCT50-1	156	170	–	177	165	181	N/A	0.96	0.95	0.94
	SCT50-2	187	205	–	212	196	215	N/A	0.97	0.95	0.95
	SCT50-3	200	219	–	228	211	232	N/A	0.96	0.95	0.94
							AVR	0.94	0.95	0.95	0.94
							S.D.	0.02	0.02	0.03	0.03

Note: “-” means not provided by the researchers; “N/A” means not available.

**Fig 9:**
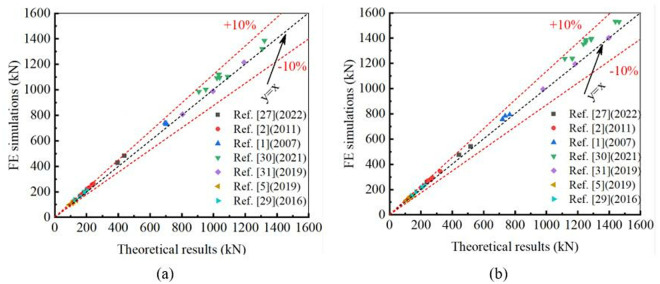
Comparison between FE simulations and theoretical predictions: (a) Yield strengths; (b) Ultimate strengths.

FE analysis was used to conduct numerical simulations for evaluating the yield strength (*N*_FE,y_) and ultimate strength (*N*_FE,u_). Comparing the theoretical results with the FE simulations, the average (AVR) and standard deviation (S.D.) of *N*_y_/*N*_FE,y_ are 0.95 and 0.03; the average and standard deviation of *N*_u_/*N*_FE,u_ are 0.94 and 0.03. Details can also be seen in Table 4. The maximum error between theoretical predictions and FE simulations is less than 10%, as shown in Fig 9. This study demonstrates that theoretical formulae can provide an accurate estimation of the structural performance of CHS stub columns strengthened with CFRP.

**Fig 10:**
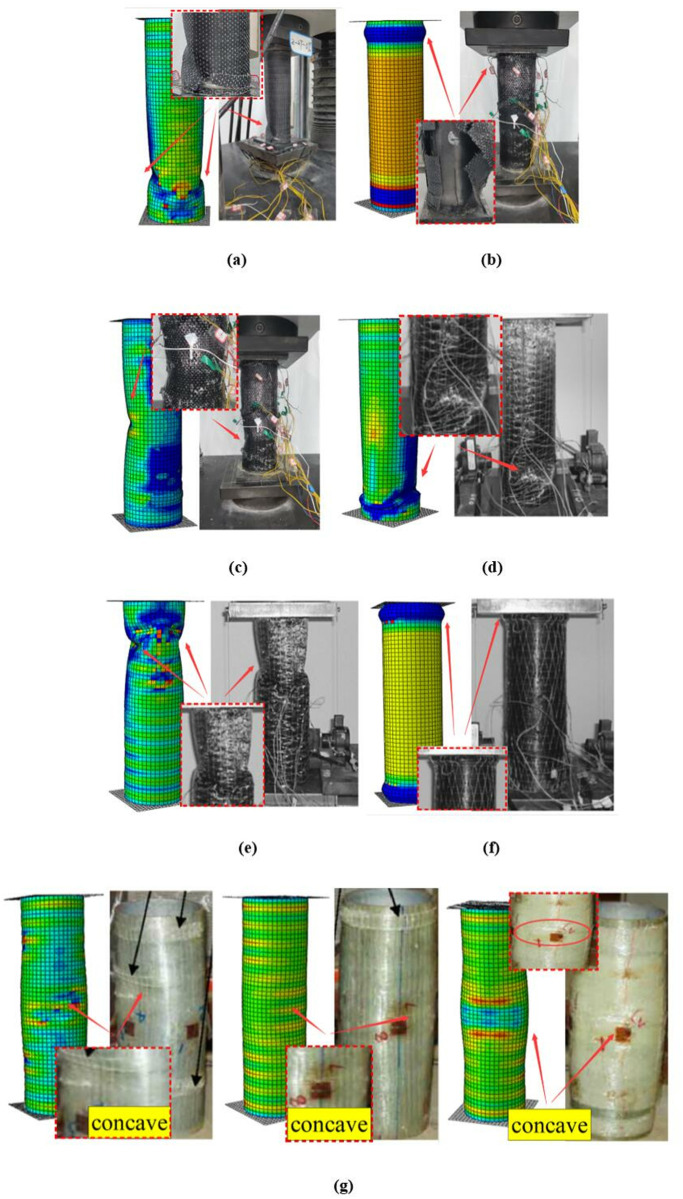
Failure modes: (a) 2-2T-0; (b) 4-1T1L-0; (c) 4-2T2L-0; (d) CF-3A; (e) CF-2A; (f) CF-2B; (g) ST-F.

It is important to mention that theoretical predictions tend to underestimate experimental results and FE simulations. This is mainly because steel tube material is treated as ideal elastic-plastic in theoretical analysis, and the effect of strain hardening on structural performance is ignored. Besides the static strengths, buckling failure modes can also be properly predicted through FE analysis in this paper, as shown in Fig 10. The reliable steps outlined in this paper for constructing the FE model can serve as a reference for similar structural analysis.

## 5. Parametric study

A parametric study is conducted based on theoretical formulas to analyze the impacts of the CHS tube thickness(*t*_s_), the diameter-to-thickness ratio(*D*/*t*_s_), the fiber configuration, the number of CFRP layers(*n*_c_), the tensile strength of CFRP(*f*_ct_), as well as the steel yield strength on the strengthening efficiency of CFRP. The value ranges for each parameter are shown in Table 5. Sixteen CHS stub columns were selected as the objects for parametric analysis, and the geometric details of these columns are presented in Table 6.

**Table 5:** Value ranges for each parameter.

Parameter	Value
**Tube thickness(*t***_**s**_)	2-11(mm)
**diameter-to-thickness ratio(*D*/*t***_**s**_)	20-57
**yield strength of steel tubes(*f***_**ys**_)	205-460(MPa)
**number of CFRP layers(*n***_**c**_)	4-10
**tensile strength of CFRP(*f***_**ct**_)	2500-4000(MPa)

**Table 6:** Geometric details of CHS stub columns.

No.	*D*/mm	*t*_s_/mm	*L*_s_/mm	*D*/*t*_s_
**1**	102.0	2.0	350.0	51.0
**2**	114.0	2.0	400.0	57.0
**3**	102.0	3.0	350.0	34.0
**4**	114.0	3.0	400.0	38.0
**5**	102.0	4.0	350.0	25.5
**6**	114.0	4.0	400.0	28.5
**7**	168.0	4.0	600.0	42.0
**8**	203.0	4.0	700.0	50.8
**9**	102.0	5.0	350.0	20.4
**10**	114.0	5.0	400.0	22.8
**11**	168.0	5.0	600.0	33.6
**12**	325.0	8.0	900.0	40.6
**13**	406.0	8.0	1500.0	50.8
**14**	406.0	9.0	1500.0	45.1
**15**	406.0	10.0	1500.0	40.6
**16**	406.0	11.0	1500.0	36.9

Note: *D*, *t*_s_ and *L*_s_ represent the outer diameter, the tube thickness and the length of steel tube respectively.

### 5.1. Effect of number of CFRP layers on strengthening efficiency

**Fig 11:**
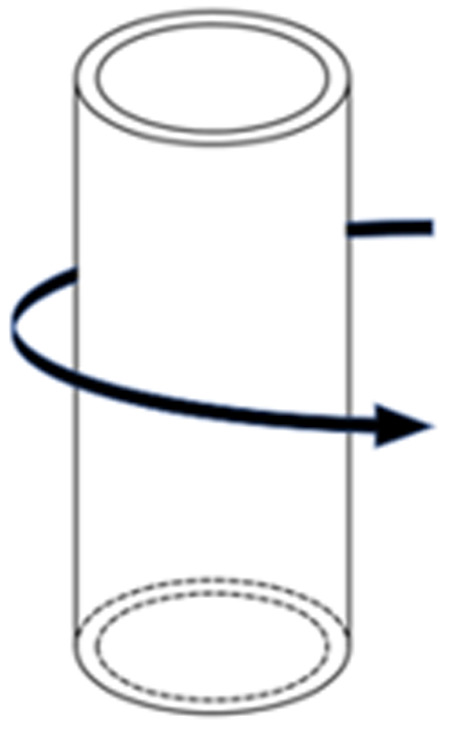
Hoop direction in steel tube.

Considering that CFRP primarily improves the ultimate strength of CHS stub columns, this section focuses only on investigating the ultimate strength of CHS stub columns strengthened with varying numbers of CFRP laminates. The number of CFRP layers varies from 1 to 4. All the CFRP laminates are bonded along the hoop direction of steel tubes, as illustrated in Fig 11. The CFRP laminate used for strengthening CHS steel tubes has a tensile strength of 3850MPa and a thickness of 0.167 mm. The CHS steel tubes discussed here have different steel yield strengths, namely 245MPa, 335MPa, 345MPa, 420MPa, and 460MPa. The diameter, thickness, and length of the steel tubes are 102 mm, 4 mm, and 350 mm, respectively.

**Fig 12:**
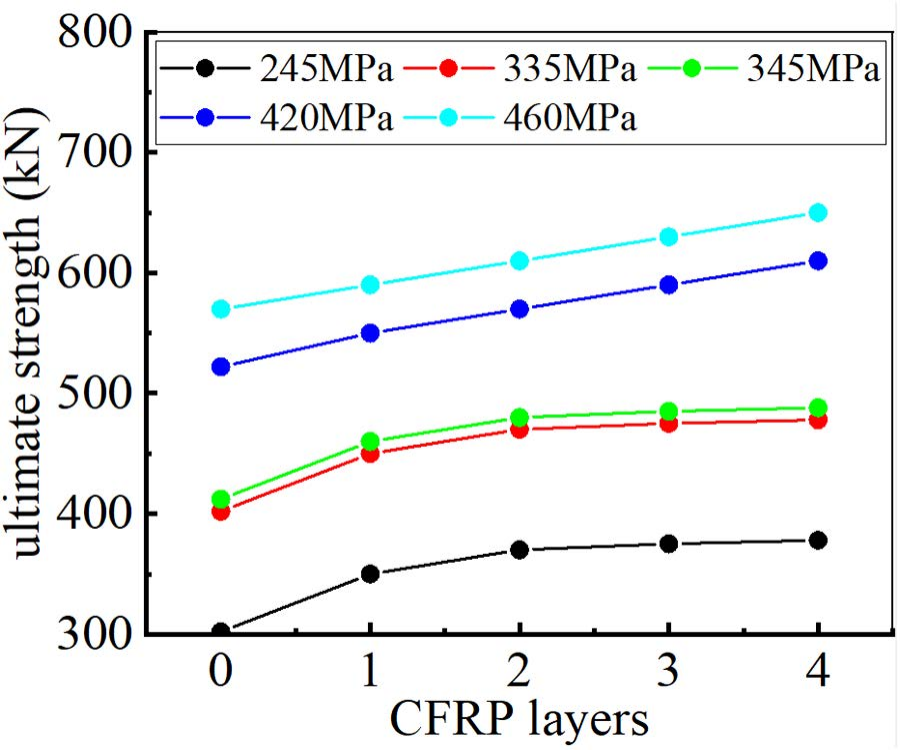
Relationship between ultimate strength and number of CFRP laminate.

The ultimate strength of CHS stub columns strengthened with varying numbers of CFRP layers is illustrated in Fig 12. As shown, the addition of CFRP layers significantly enhances the ultimate strength of these columns. However, the rate of improvement decreases when the number of CFRP layers exceeds a certain threshold. This implies that adding more CFRP layers does not continuously enhance the ultimate strength. No significant enhancement in ultimate strength is observed when the number of CFRP layers exceeds 4 for steel tubes with yield strengths of 245MPa, 335MPa, and 345MPa, as illustrated in Fig 12.

In section 2.3, the confinement coefficient *λ* is derived to determine the optimal number of CFRP laminates bonded on the surface of the steel tube. When *λ* ≤ 1.155, both construction cost and strengthening efficiency requirements are satisfied. Table 7 presents *λ* values for different CFRP layer counts.

**Table 7:** The confinement coefficient *λ* at different numbers of CFRP layers.

Yield strength (MPa)	confinement coefficient *λ*			
	1 layer	2 layers	3 layers	4 layers
**245**	0.41	0.82	1.21	1.64
**335**	0.30	0.61	0.90	1.20
**345**	0.29	0.58	0.87	1.16
**420**	0.24	0.48	0.71	0.95
**460**	0.22	0.44	0.65	0.87

**Fig 13:**
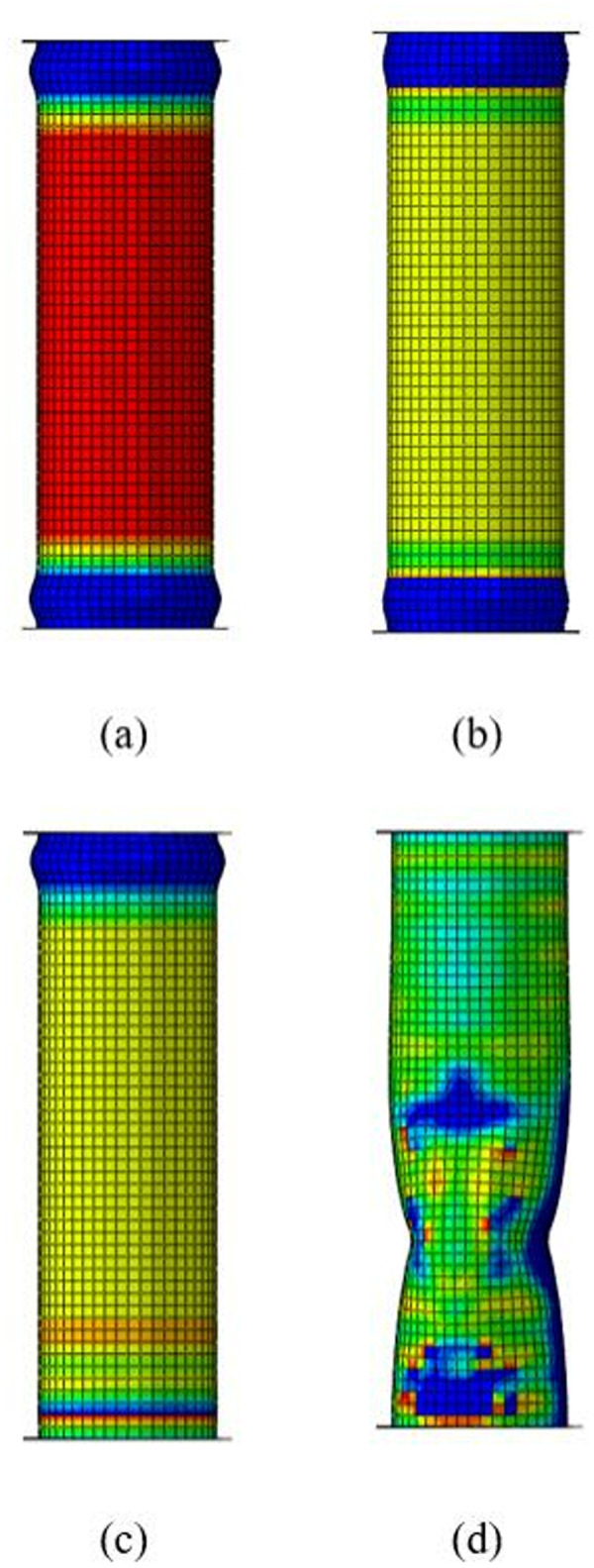
Buckling modes under different numbers of CFRP layers: (a) 1 layer; (b) 2 layers; (c) 3 layers; (d) 4 layers.

For steel tubes with lower yield strengths, such as 245MPa, 335MPa, and 345MPa, discussed in this section, the confinement coefficient *λ* exceeds 1.155 when the number of CFRP layers reaches 4. The buckling modes of steel tubes with a yield strength of 335MPa are illustrated in Fig 13. When the number of CFRP layers reaches 4, the inward deformation begins to dominate the buckling behavior of the steel tube with a yield strength of 335MPa, and the CFRP layers offer no significant restraint against inward buckling. Therefore, continuously increasing the number of CFRP layers beyond 4 does not contribute to the structural performance in this situation (*λ* > 1.155).

### 5.2. Effect of CFRP configuration on strengthening efficiency

**Fig 14:**
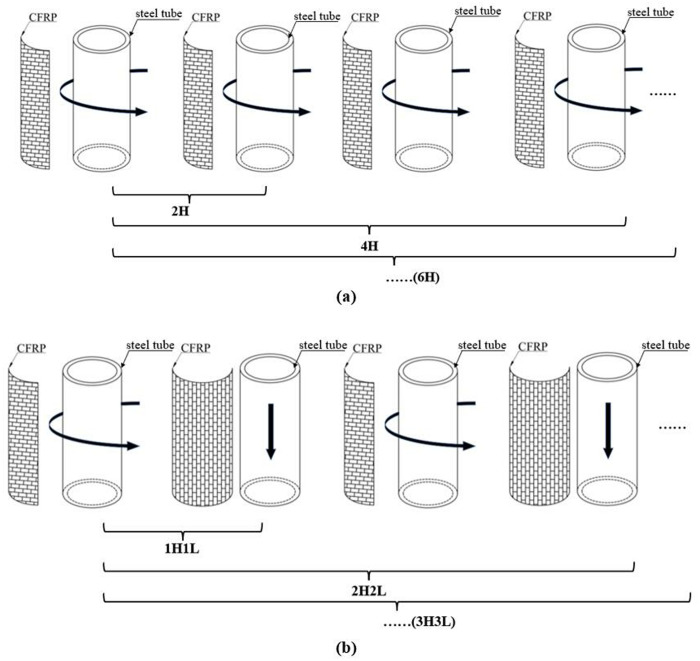
Strengthening schemes for steel tube: (a) Hoop; (b) Hoop and longitudinal.

To analyze the influence of CFRP configuration on strengthening efficiency, three groups of CFRP strengthening schemes were designed, as detailed in Table 8 and Fig 14. Different schemes were utilized to strengthen the CHS stub columns, and a comparison of their static strength before and after strengthening was conducted to ascertain the impact of CFRP configuration on strengthening efficiency.

**Table 8:** Strengthening schemes.

Group	Configuration	
	Hoop	Hoop and longitudinal
**1**	2H	1H1L
**2**	4H	2H2L
**3**	6H	3H3L

Similar to the section 5.1, this section focuses on investigating the ultimate strength of CHS stub columns strengthened with CFRP bonded in different configurations. The CHS steel tube has an outer diameter (*D*) of 102mm, a tube thickness (*t*_s_) of 4 mm and a length (*L*_s_) of 350 mm. The yield strength, the elastic modulus and the Poisson’s ratio of the steel tube are 335MPa, 206GPa and 0.28, respectively.

The CHS stub columns were reinforced according to the designed strengthening schemes, and the ultimate strength of the reinforced CHS stub columns is listed in Table 9. With the same number of CFRP layers, the hoop bonded CFRP results in a more significant improvement in the ultimate strength of CHS stub columns.

**Table 9:** Ultimate strength of CHS stub columns after CFRP strengthening.

Group	Configuration	Ultimate strength kN
**1**	2H	481.2
	1H1L	462.6
**2**	4H	541.5
	2H2L	482.2
**3**	6H	581.7
	3H3L	502.2

**Fig 15:**
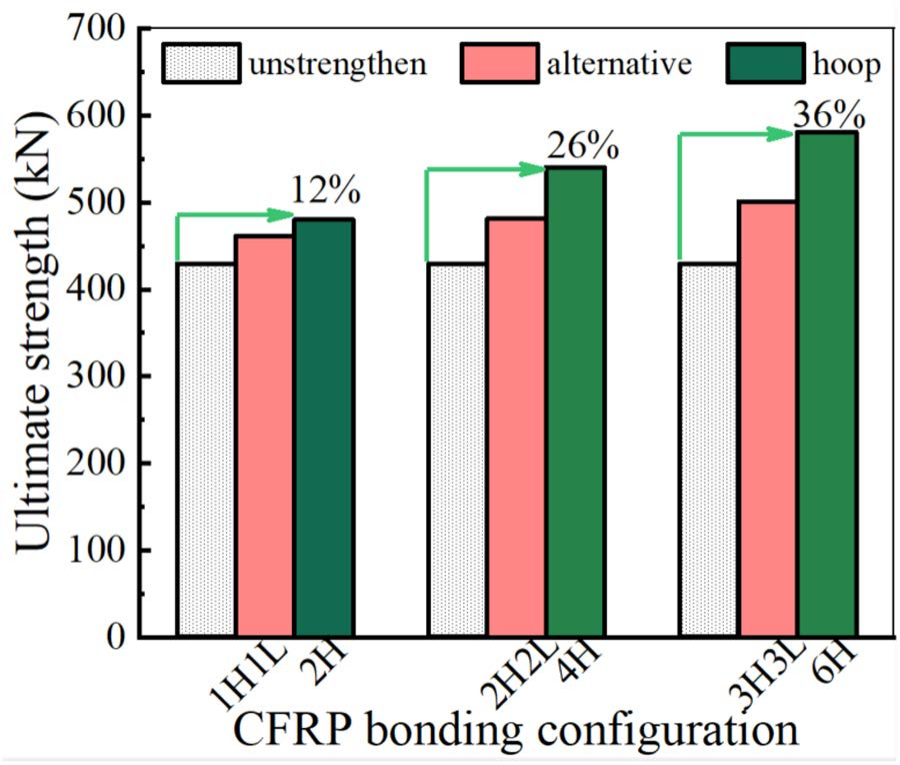
Effect of bonding configuration on strengthening efficiency.

The fracture strain of CFRP in its primary direction is much greater than that in the secondary direction. The carbon fibers in primary direction under tension state can make their material properties be fully utilized and better strengthening effects be achieved. Hoop bonding enables carbon fibers to be tensioned in their primary direction, while longitudinal bonding makes carbon fibers in a state of compression, limiting their contribution to strengthening. When comparing the different bonding configurations, hoop bonding can better utilize the material properties of CFRP and is more effective in enhancing the ultimate strength of CHS stub columns. The ultimate strength of CHS stub columns can be increased by 36% with 6 layers of hoop CFRP laminates, whereas with 6 layers of CFRP laminates bonded in an alternating hoop and longitudinal pattern, the ultimate strength of CHS stub columns can only be increased by 16%, as shown in Fig 15.

### 5.3. Effect of CFRP tensile strength on strengthening efficiency

**Fig 16:**
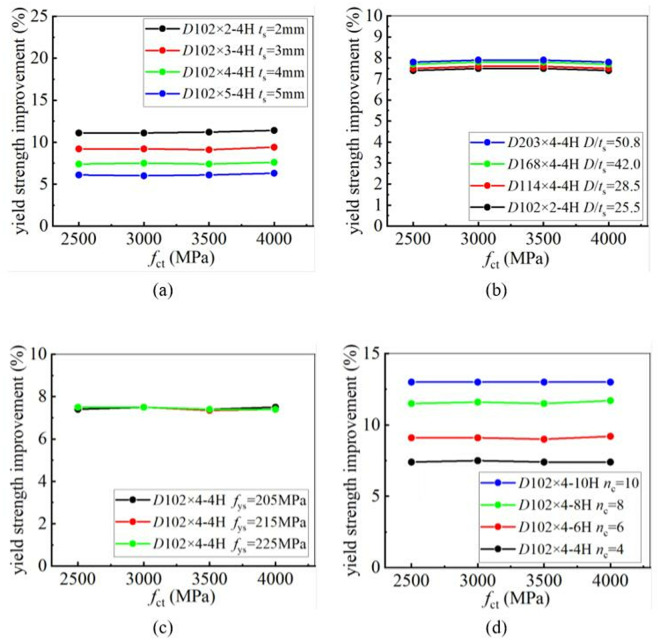
The influence of CFRP tensile strength on yield strength improvement under different conditions: (a) Different tube thickness; (b) Different diameter-to-thickness ratio; (c) Different yield strength; (d) Different number of CFRP layer.

**Fig 17:**
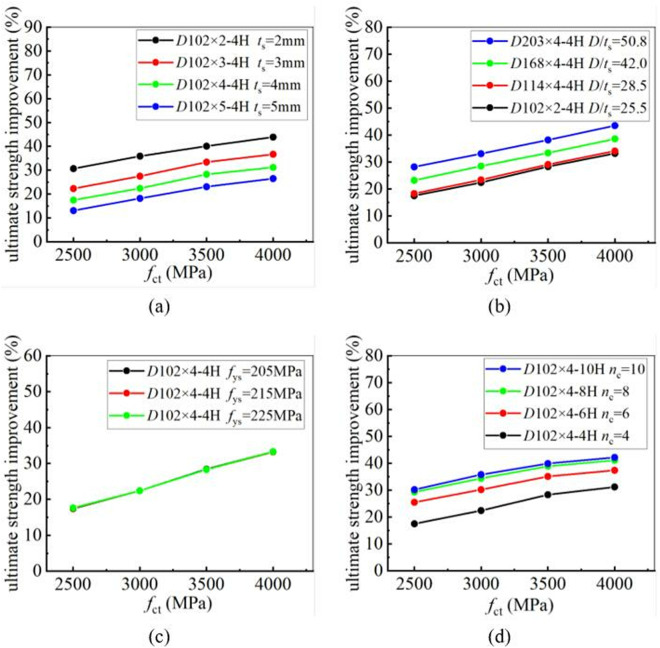
The influence of CFRP tensile strength on ultimate strength improvement under different conditions: (a) Different tube thickness; (b) Different diameter-to-thickness ratio; (c) Different yield strength; (d) Different number of CFRP layer.

To analyze the influence of CFRP tensile strength on the strengthening efficiency, 4 grades of CFRP with different tensile strengths were selected for a parametric study. The tensile strengths are 2500 MPa, 3000 MPa, 3500 MPa, and 4000 MPa, respectively. The yield and ultimate strengths of CFRP-strengthened CHS stub columns, reinforced with CFRP laminates having different tensile strengths, were calculated. The calculation results showed that the tensile strength has no influence on the improvement of the yield strength of CHS stub columns. CFRP laminates with different tensile strengths provide almost the same level of enhancement. However, CFRP laminates with higher tensile strengths can provide better enhancement in the ultimate strength of CHS stub columns. This conclusion remains unchanged, regardless of variations in the tube thickness, the diameter-to-thickness ratio of the steel tube, the number of CFRP layers, and the yield strength of the steel tube. Figs 16 and 17 clearly demonstrate the relationship between CFRP tensile strength and strengthening efficiency. In these figures, “4H, 6H, 8H, and 10H” indicate that four, six, eight, or ten layers of CFRP are placed along the hoop direction.

### 5.4. Effect of diameter-to-thickness ratio on strengthening efficiency

**Fig 18:**
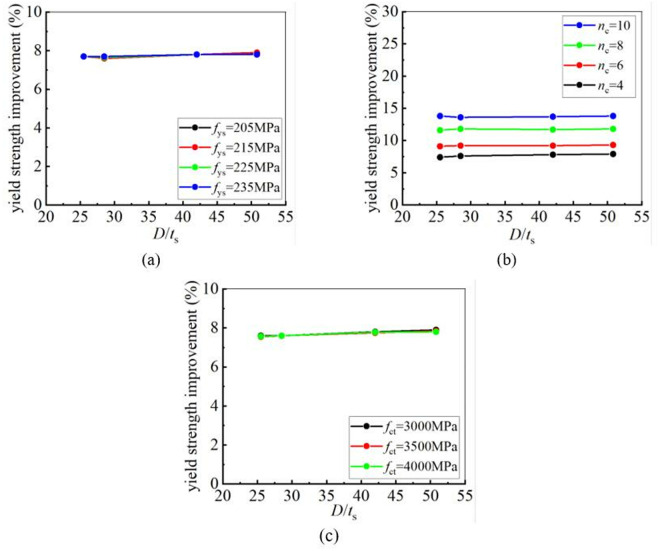
The influence of diameter-to-thickness ratio on yield strength improvement under different conditions: (a) Different yield strength; (b) Different number of CFRP layers; (c) Different CFRP tensile strength.

**Fig 19:**
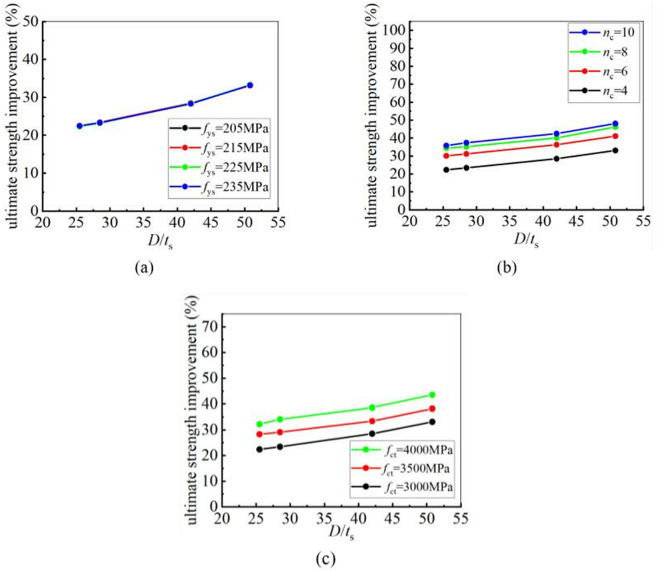
The influence of diameter-to-thickness ratio on ultimate strength improvement under different conditions: (a) Different yield strength; (b) Different number of CFRP layers; (c) Different CFRP tensile strength.

To analyze the influence of the diameter-to-thickness ratio of steel tubes on the strengthening efficiency of CFRP, four CHS steel tubes with diameter-to-thickness ratios of 25.5(*D*102 × 4), 28.5(*D*114 × 4), 42.0(*D*168 × 4), and 50.8(*D*203 × 4) were selected from Table 6 for a parametric study. The yield and ultimate strengths of CFRP-strengthened CHS stub columns with different diameter-to-thickness ratios were calculated. The calculation results revealed that the diameter-to-thickness ratio has no influence on the improvement in yield strength but significantly affects the enhancement in ultimate strength. As the diameter-to-thickness ratio increases, the improvement in ultimate strength becomes greater. This conclusion remains unchanged regardless of variations in the yield strength of the steel tube, the number of CFRP layers and the tensile strength of CFRP. Figs 18 and 19 clearly illustrate the relationship between the diameter-to-thickness ratio and the strengthening efficiency of CFRP.

### 5.5. Effect of tube thickness on strengthening efficiency

**Fig 20:**
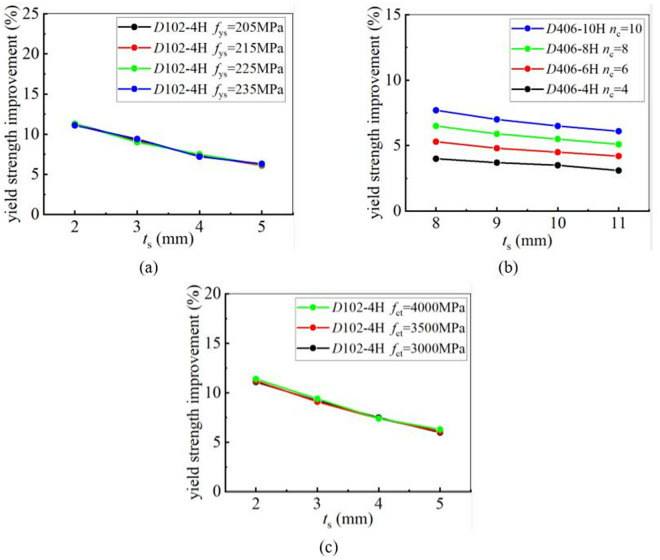
The influence of tube thickness on yield strength improvement under different conditions: (a) Different yield strength; (b) Different number of CFRP layers; (c) Different CFRP tensile strength.

**Fig 21:**
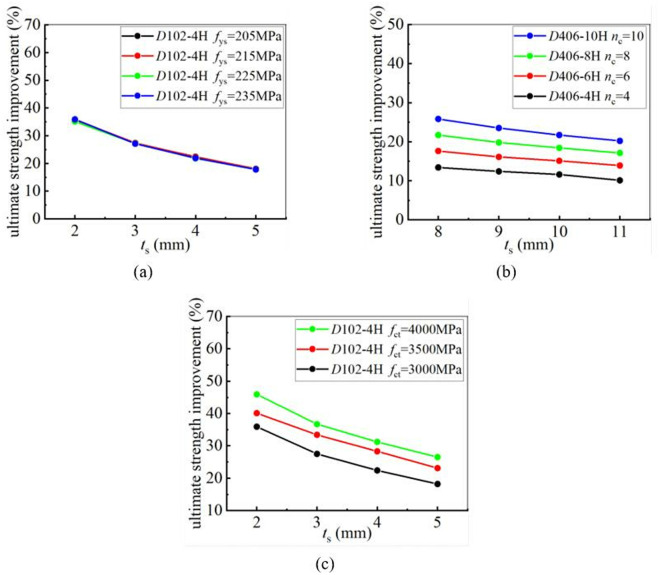
The influence of tube thickness on ultimate strength improvement under different conditions: (a) Different yield strength; (b) Different number of CFRP layers; (c) Different CFRP tensile strength.

To investigate the influence of the tube thickness on the strengthening efficiency of CFRP, eight steel tubes with varying thicknesses were selected from Table 6 for a parametric study. The yield and ultimate strengths of CFRP-strengthened CHS stub columns with varying tube thickness were calculated and listed in Figs 20 and 21. In Figs 20 and 21, “4H, 6H, 8H and 10H” indicate that four, six, eight or ten layers of CFRP are placed along the hoop direction. It is clear that the improvement in both the yield and ultimate strengths of CHS stub columns decreases as the steel tube thickness increases. The greater the tube thickness, the smaller the enhancement in the static strengths. This conclusion remains unchanged regardless of variations in the yield strength of steel tube, the number of CFRP layers and the tensile strength of CFRP.

### 5.6. Effect of steel yield strength on strengthening efficiency

**Fig 22:**
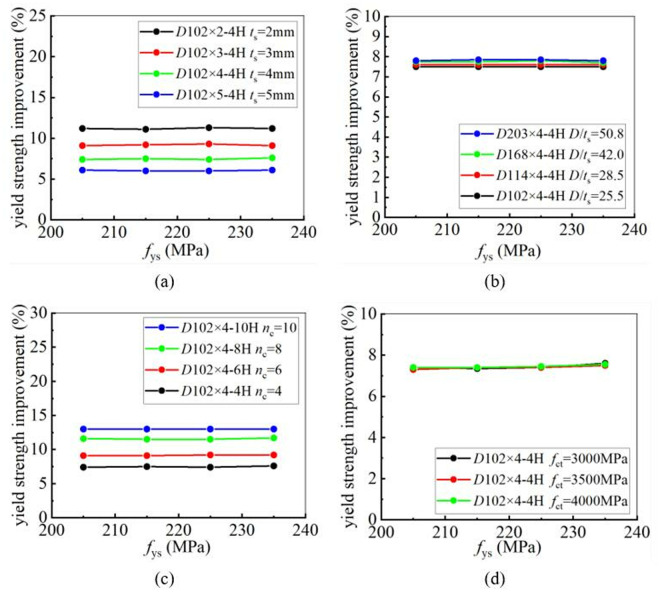
The influence of steel material performance on yield strength improvement under different conditions: (a) Different tube thickness; (b) Different diameter-to-thickness ratio; (c) Different number of CFRP layers; (d) Different CFRP tensile strength.

**Fig 23:**
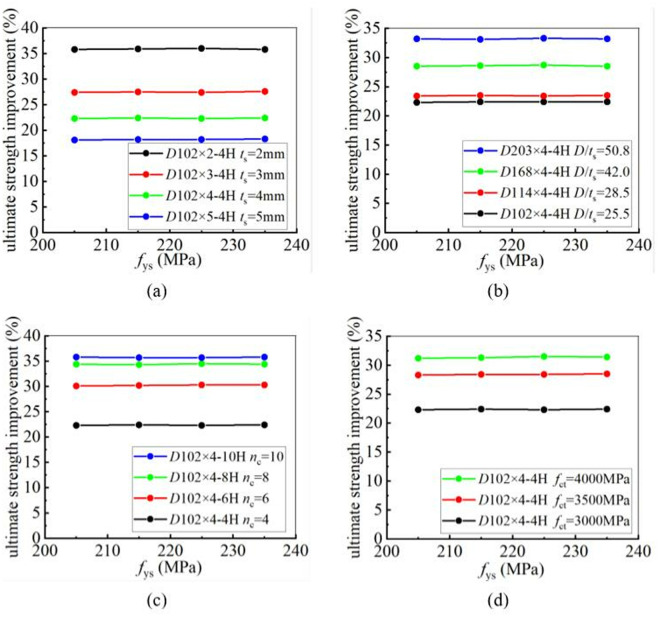
The influence of steel material performance on ultimate strength improvement under different conditions: (a) Different tube thickness; (b) Different diameter-to-thickness ratio; (c) Different number of CFRP layers; (d) Different CFRP tensile strength.

To determine the influence of the yield strength of steel tubes on the CFRP strengthening efficiency, four steel tubes with different yield strengths, namely 205 MPa, 215 MPa, 225 MPa, and 235 MPa, were selected for a parametric study. The yield and ultimate strengths of CFRP-strengthened CHS stub columns were calculated. The results demonstrated that the steel yield strength has no significant influence on the improvement of the static strengths of CFRP-strengthened CHS stub columns. This conclusion remains unchanged, regardless of variations in the tube thickness, the diameter-to-thickness ratio, the number of CFRP layers, or the tensile strength of CFRP. Figs 22 and 23 clearly demonstrate the relationship between steel yield strength and CFRP strengthening efficiency. In Figs 22 and 23, “4H, 6H, 8H and 10H” indicate that four, six, eight or ten layers of CFRP are placed along hoop direction.

## 6. Conclusion

Both the theoretical analysis and the numerical simulation have been performed in this study to investigate the static strengths of CHS stub columns strengthened with CFRP. Based on these investigations, the following conclusions can be drawn:

(1) A series of calculation formulae are derived to predict the structural performance of CFRP-strengthened CHS stub columns. The accuracy of these formulae is verified by comparing the theoretical predictions with the experimental results and FE simulations separately. The maximum errors among them are within 10%.
(2) The confinement coefficient *λ* used to evaluate the confinement efficiency of CFRP on CHS stub columns is derived. The threshold of *λ* is found to be no greater than 1.155. Increasing the number of CFRP layers can effectively improve the static strength as long as *λ* ≤ 1.155. Otherwise, continuously increasing the number of CFRP layers only increases the construction cost meaninglessly.
(3) Hoop-placed CFRP provides a better strengthening effect for CHS stubs compared to the alternating hoop and longitudinal CFRP pattern. Applying 6 layers of hoop CFRP can result in a 36% enhancement in the specimen’s load bearing capacity, while 6 layers of alternating hoop and longitudinal placed CFRP only provide a 16% improvement for the same CHS tube.
(4) Both the diameter-to-thickness ratio and the tensile strength of CFRP have almost no influence on the improvement of yield strength of CHS stub columns but significantly affect the ultimate strength. With the increase in diameter-to-thickness ratio and tensile strength of CFRP, the improvement in ultimate strength becomes greater.
(5) The improvement in static strength of CHS stub columns decreases as the steel tube thickness increases. The greater the tube thickness, the smaller the enhancement in static strength. The steel yield strength has no effect on the strengthening efficiency of CFRP, and the strengthening efficiency is mainly affected by the geometry of CHS steel tube as well as the bonding configuration and the material properties of CFRP laminate.

## 7. Future prospect

Researchers have been investigating the mechanical behavior of CFRP-strengthened CHS steel tubes for many years. They have confirmed the effectiveness and feasibility of using CFRP to strengthen steel tubular structures and identified the crucial factors influencing the strengthening efficiency. However, there are still some limitations in this field, and more attention should be paid to them in future research.

1. Currently, most studies focus on CHS steel tubes without pre-existing loads. However, for specific structures like offshore jacket platforms and offshore wind turbines, it is often impractical to unload these structures before strengthening. Consequently, the strengthening work has to be carried out under preloading conditions in practice. There are gaps between research findings and practical applications. The specific impact of initial loading on CFRP-strengthened tubular structures needs to be elucidated in future studies. The effectiveness of CFRP-strengthening on preloaded tubular structures is a key research focus in the future.
2. Existing research mainly focuses on the static and seismic performance of CHS steel tubes. Further discussions on the mechanical behavior of such members under impact loading or blast loading are needed in future studies.

The CFRP-strengthening technique has significantly propelled the development of strengthened steel tubular members and provided engineers with an additional option to solve practical problems. With the advent of materials like smart-memory alloys, pre-stressed CFRP for strengthening steel structures with complex geometries has become feasible. Additionally, with the assistance of artificial intelligence (AI), the design of novel composite materials and structures, the refinement of theoretical formulations, and specifications, as well as the development of low-carbon CFRP production processes, are expected to undergo substantial improvements in the future.

## Supporting information

S1 Data Load bearing capacity. (DOCX)

S1 Table Model details. (DOCX)

S2 Table Material properties of CHS steel tube. (DOCX)

S3 Table Mechanical properties and geometric details of FRP laminate. (DOCX)

S4 Table Comparisons of load bearing capacity. (DOCX)

S5 Table Value ranges for each parameter. (DOCX)

S6 Table Geometric details of CHS stub columns. (DOCX)

S7 Table The confinement coefficient *λ* at different numbers of CFRP layers. (DOCX)

S8 Table Strengthening schemes. (DOCX)

S9 Table Ultimate strength of CHS stub columns after CFRP strengthening. (DOCX)
