# Supplementary material for: Static strengths of circular hollow section stub column strengthened with carbon fiber reinforced polymer
Source: PLoS One. 2025 Aug 1;20(8):e0328047. doi: 10.1371/journal.pone.0328047 (PMC12316273; doi:10.1371/journal.pone.0328047)
Supplement: S1 Table — (DOCX) [file pone.0328047.s002.docx]

**Table 1. Model details**

| **Ref.** | **ID** | ***D***  **mm** | ***t***  **mm** | ***L***  **mm** | ***D*/*t*** | **CFRP** |
| --- | --- | --- | --- | --- | --- | --- |
| **[27]** | 4-1T1L-0 | 89.0 | 4.0 | 300.0 | 22.3 | 1H1L |
|  | 4-2T2L-0 | 89.0 | 4.0 | 300.0 | 22.3 | 2H2L |
|  | 2-2T-0 | 85.0 | 2.0 | 300.0 | 42.5 | 2H |
| **[2]** | CF-1A | 87.2 | 2.3 | 279.0 | 37.9 | 1H1L |
|  | CF-1B | 87.2 | 2.3 | 279.0 | 37.9 | 2H2L |
|  | CF-2A | 86.4 | 1.9 | 276.5 | 45.5 | 1H1L |
|  | CF-2B | 86.4 | 2.0 | 276.5 | 43.2 | 2H2L |
|  | CF-3A | 85.7 | 1.6 | 274.2 | 53.6 | 1H1L |
| **[1]** | ST-F1 | 166.0 | 4.2 | 450.0 | 39.5 | 1H |
|  | ST-F2 | 165.0 | 4.2 | 450.0 | 39.3 | 2H |
|  | ST-F3 | 165.0 | 4.2 | 450.0 | 39.3 | 3H |
| **[30]** | S168L5T3C | 168.0 | 5.0 | 500.0 | 33.6 | 3H |
|  | S168L5T5C | 168.0 | 5.0 | 500.0 | 33.6 | 3H |
|  | S168L5T7C | 168.0 | 5.0 | 500.0 | 33.6 | 3H |
|  | S140L5T3C | 140.0 | 5.0 | 500.0 | 28.0 | 3H |
|  | S140L5T5C | 140.0 | 5.0 | 500.0 | 28.0 | 3H |
|  | S140L5T7C | 140.0 | 5.0 | 500.0 | 28.0 | 3H |
|  | S140L8T3C | 140.0 | 5.0 | 800.0 | 28.0 | 3H |
|  | S140L8T5C | 140.0 | 5.0 | 800.0 | 28.0 | 3H |
|  | S140L8T7C | 140.0 | 5.0 | 800.0 | 28.0 | 3H |
| **[31]** | S-1A-C | 168.0 | 5.0 | 500.0 | 33.6 | 3H3L |
|  | S-2A-C | 180.0 | 5.0 | 1500 | 36.0 | 3H3L |
|  | S-2A-C1 | 180.0 | 5.0 | 1500 | 36.0 | 3H3L |
| **[5]** | S1-20-A1 | 58.0 | 1.0 | 200.0 | 58.0 | 1H |
|  | S1-20-A2 | 58.0 | 1.0 | 200.0 | 58.0 | 2H |
|  | S1-20-A3 | 58.0 | 1.0 | 200.0 | 58.0 | 3H |
|  | S1.5-20-A1 | 58.0 | 1.5 | 200.0 | 38.7 | 1H |
|  | S2-20-A1 | 58.0 | 2.0 | 200.0 | 29.0 | 1H |
|  | S2-20-A2 | 58.0 | 2.0 | 200.0 | 29.0 | 2H |
| **[29]** | SCT50-0 | 49.7 | 3.1 | 150.0 | 16.0 | - |
|  | SCT50-1 | 49.7 | 3.1 | 150.0 | 16.0 | 1H1L |
|  | SCT50-2 | 49.7 | 3.1 | 150.0 | 16.0 | 2H2L |
|  | SCT50-3 | 49.7 | 3.1 | 150.0 | 16.0 | 3H3L |

Note: '-' means that specimen 'SCT50-0' has not been strengthened with CFRP.
