# Supplementary material for: Static strengths of circular hollow section stub column strengthened with carbon fiber reinforced polymer
Source: PLoS One. 2025 Aug 1;20(8):e0328047. doi: 10.1371/journal.pone.0328047 (PMC12316273; doi:10.1371/journal.pone.0328047)
Supplement: S2 Table — (DOCX) [file pone.0328047.s003.docx]

**Table 2. Material properties of CHS steel tube**

| **Ref.** | **ID** | ***E*_s_**  **GPa** | ***f*_y_**  **MPa** | ***f*_u_**  **MPa** | ***μ*_s_** |
| --- | --- | --- | --- | --- | --- |
| **[27]** | 4-1T1L-0 | 200.3 | 335.0 | 454.0 | 0.28 |
|  | 4-2T2L-0 | 200.3 | 335.0 | 454.0 | 0.28 |
|  | 2-2T-0 | 200.3 | 335.0 | 454.0 | 0.28 |
| **[2]** | CF-1A | 209.5 | 455.0 | 507.0 | 0.3 |
|  | CF-1B | 209.5 | 455.0 | 507.0 | 0.3 |
|  | CF-2A | 209.5 | 455.0 | 507.0 | 0.3 |
|  | CF-2B | 209.5 | 455.0 | 507.0 | 0.3 |
|  | CF-3A | 209.5 | 455.0 | 507.0 | 0.3 |
| **[1]** | ST-F1 | 201.0 | 333.0 | 370.0 | 0.34 |
|  | ST-F2 | 201.0 | 333.0 | 370.0 | 0.34 |
|  | ST-F3 | 201.0 | 333.0 | 370.0 | 0.34 |
| **[30]** | S168L5T3C | 190.0 | 408.0 | 604.0 | 0.28 |
|  | S168L5T5C | 195.0 | 428.0 | 611.0 | 0.28 |
|  | S168L5T7C | 199.0 | 393.0 | 554.0 | 0.28 |
|  | S140L5T3C | 207.0 | 442.0 | 612.0 | 0.28 |
|  | S140L5T5C | 215.0 | 472.0 | 660.0 | 0.28 |
|  | S140L5T7C | 207.0 | 414.0 | 581.0 | 0.28 |
|  | S140L8T3C | 207.0 | 442.0 | 612.0 | 0.28 |
|  | S140L8T5C | 215.0 | 472.0 | 660.0 | 0.28 |
|  | S140L8T7C | 207.0 | 414.0 | 581.0 | 0.28 |
| **[31]** | S-1A-C | 200.0 | 345.5 | 499.0 | 0.3 |
|  | S-2A-C | 200.0 | 345.5 | 499.0 | 0.3 |
|  | S-2A-C1 | 200.0 | 345.5 | 499.0 | 0.3 |
| **[5]** | S1-20-A1 | 196.0 | 370.0 | 549.0 | 0.3 |
|  | S1-20-A2 | 196.0 | 370.0 | 549.0 | 0.3 |
|  | S1-20-A3 | 196.0 | 370.0 | 549.0 | 0.3 |
|  | S1.5-20-A1 | 208.0 | 272.0 | 410.0 | 0.3 |
|  | S2-20-A1 | 208.0 | 272.0 | 410.0 | 0.3 |
|  | S2-20-A2 | 208.0 | 272.0 | 410.0 | 0.3 |
| **[29]** | SCT50-0 | 70.3 | 291.0 | 295.0 | 0.36 |
|  | SCT50-1 | 70.3 | 291.0 | 295.0 | 0.36 |
|  | SCT50-2 | 70.3 | 291.0 | 295.0 | 0.36 |
|  | SCT50-3 | 70.3 | 291.0 | 295.0 | 0.36 |
