# Supplementary material for: Static strengths of circular hollow section stub column strengthened with carbon fiber reinforced polymer
Source: PLoS One. 2025 Aug 1;20(8):e0328047. doi: 10.1371/journal.pone.0328047 (PMC12316273; doi:10.1371/journal.pone.0328047)
Supplement: S3 Table — (DOCX) [file pone.0328047.s004.docx]

**Table 3. Mechanical properties and geometric details of FRP laminate**

| **Ref.** | ***f*_ct_**  **MPa** | ***E*_ct_**  **GPa** | ***ε*_c_** | ***f*_cc_**  **MPa** | ***E*_cc_**  **GPa** | ***t*_FRP_**  **mm** |
| --- | --- | --- | --- | --- | --- | --- |
| **[27]** | 3720 | 228 | 0.016 | 92.6 | 3.03 | 0.167 |
| **[1]** | 1825.5 | 80.1 | 0.0228 | N/A | N/A | 0.170 |
| **[2]** | 1830 | 230 | N/A | 32 | 1.9 | 0.176 |
| **[30]** | 3720 | 228 | 0.016 | N/A | N/A | 0.167 |
| **[31]** | 2650 | 238 | 0.014 | N/A | N/A | 0.167 |
| **[5]** | 1400 | 79 | N/A | 132 | 5.5 | 0.250 |
| **[29]** | 2034 | 71.5 | N/A | 10.7 | N/A | 0.650 |

Note: 'N/A' means that relevant information is not provided by the previous research.
