# Supplementary material for: Static strengths of circular hollow section stub column strengthened with carbon fiber reinforced polymer
Source: PLoS One. 2025 Aug 1;20(8):e0328047. doi: 10.1371/journal.pone.0328047 (PMC12316273; doi:10.1371/journal.pone.0328047)
Supplement: S4 Table — (DOCX) [file pone.0328047.s005.docx]

**Table 4. Comparisons of load bearing capacity**

| **Ref.** | **ID** | ***N***_y_  /kN | ***N***_u_  /kN | ***N***_EX,y_  /kN | ***N***_EX,u_  /kN | ***N***_FE,y_  /kN | ***N***_FE,u_  /kN | $\frac{N_{\text{y}}}{N_{\text{EX,y}}}$ | $\frac{N_{\text{u}}}{N_{\text{EX,u}}}$ | $\frac{N_{\text{y}}}{N_{\text{FE,y}}}$ | $\frac{N_{\text{u}}}{N_{\text{FE,u}}}$ |
| --- | --- | --- | --- | --- | --- | --- | --- | --- | --- | --- | --- |
| **[27]** | 4-1T1L-0 | 394 | 442 | 429 | 472 | 430 | 477 | 0.92 | 0.94 | 0.92 | 0.93 |
|  | 4-2T2L-0 | 437 | 516 | 479 | 535 | 485 | 543 | 0.91 | 0.96 | 0.91 | 0.95 |
|  | 2-2T-0 | 182 | 236 | 190 | 256 | 193 | 260 | 0.96 | 0.92 | 0.94 | 0.91 |
| **[2]** | CF-1A | 235 | 273 | 248 | 299 | 255 | 293 | 0.95 | 0.91 | 0.92 | 0.93 |
|  | CF-1B | 244 | 324 | 261 | 341 | 262 | 343 | 0.93 | 0.95 | 0.93 | 0.94 |
|  | CF-2A | 203 | 244 | 223 | 267 | 222 | 269 | 0.91 | 0.91 | 0.91 | 0.91 |
|  | CF-2B | 215 | 263 | 236 | 281 | 231 | 277 | 0.91 | 0.94 | 0.93 | 0.95 |
|  | CF-3A | 167 | 201 | 183 | 214 | 177 | 213 | 0.91 | 0.94 | 0.94 | 0.94 |
| **[1]** | ST-F1 | 701 | 720 | 724 | 740 | 729 | 757 | 0.97 | 0.97 | 0.96 | 0.95 |
|  | ST-F2 | 703 | 737 | 731 | 771 | 737 | 783 | 0.96 | 0.96 | 0.95 | 0.94 |
|  | ST-F3 | 695 | 766 | 733 | 782 | 740 | 794 | 0.95 | 0.98 | 0.94 | 0.96 |
| **[30]** | S168L5T3C | 1307 | 1463 | 1385 | 1541 | 1322 | 1529 | 0.94 | 0.95 | 0.99 | 0.96 |
|  | S168L5T5C | 1321 | 1440 | 1401 | 1500 | 1385 | 1531 | 0.94 | 0.96 | 0.95 | 0.94 |
|  | S168L5T7C | 1089 | 1285 | 1154 | 1324 | 1103 | 1389 | 0.94 | 0.97 | 0.99 | 0.93 |
|  | S140L5T3C | 1034 | 1251 | 1096 | 1330 | 1121 | 1385 | 0.94 | 0.94 | 0.92 | 0.90 |
|  | S140L5T5C | 1033 | 1251 | 1097 | 1331 | 1118 | 1368 | 0.94 | 0.94 | 0.92 | 0.91 |
|  | S140L5T7C | 952 | 1165 | 1000 | 1214 | 1003 | 1238 | 0.95 | 0.96 | 0.95 | 0.94 |
|  | S140L8T3C | 1039 | 1288 | 1081 | 1342 | 1095 | 1396 | 0.96 | 0.96 | 0.95 | 0.92 |
|  | S140L8T5C | 1022 | 1236 | 1083 | 1301 | 1092 | 1353 | 0.94 | 0.95 | 0.94 | 0.91 |
|  | S140L8T7C | 908 | 1117 | 963 | 1201 | 985 | 1237 | 0.94 | 0.93 | 0.92 | 0.90 |
| **[31]** | S-1A-C | 1193 | 1398 | 1232 | 1410 | 1215 | 1402 | 0.97 | 0.99 | 0.98 | 0.99 |
|  | S-2A-C | 805 | 979 | 816 | 990 | 808 | 995 | 0.99 | 0.99 | 0.99 | 0.98 |
|  | S-2A-C1 | 997 | 1183 | 1000 | 1200 | 989 | 1195 | 0.99 | 0.99 | 1.00 | 0.99 |
| **[5]** | S1-20-A1 | 90 | 105 | - | 110 | 98 | 115 | N/A | 0.95 | 0.92 | 0.91 |
|  | S1-20-A2 | 108 | 123 | - | 132 | 110 | 131 | N/A | 0.93 | 0.98 | 0.94 |
|  | S1-20-A3 | 120 | 136 | - | 145 | 127 | 149 | N/A | 0.94 | 0.94 | 0.91 |
|  | S1.5-20-A1 | 116 | 129 | - | 135 | 121 | 142 | N/A | 0.96 | 0.96 | 0.91 |
|  | S2-20-A1 | 119 | 144 | - | 154 | 133 | 157 | N/A | 0.94 | 0.90 | 0.92 |
|  | S2-20-A2 | 136 | 153 | - | 161 | 132 | 155 | N/A | 0.95 | 1.03 | 0.99 |
| **[29]** | SCT50-0 | 128 | 141 | - | 148 | 137 | 150 | N/A | 0.95 | 0.93 | 0.94 |
|  | SCT50-1 | 156 | 170 | - | 177 | 165 | 181 | N/A | 0.96 | 0.95 | 0.94 |
|  | SCT50-2 | 187 | 205 | - | 212 | 196 | 215 | N/A | 0.97 | 0.95 | 0.95 |
|  | SCT50-3 | 200 | 219 | - | 228 | 211 | 232 | N/A | 0.96 | 0.95 | 0.94 |
|  | | | | | | | AVR | 0.94 | 0.95 | 0.95 | 0.94 |
|  |  |  |  |  |  |  | S.D. | 0.02 | 0.02 | 0.03 | 0.03 |

Note: "-" means not provided by researchers; "N/A" means not available.
