# Supplementary material for: Static strengths of circular hollow section stub column strengthened with carbon fiber reinforced polymer
Source: PLoS One. 2025 Aug 1;20(8):e0328047. doi: 10.1371/journal.pone.0328047 (PMC12316273; doi:10.1371/journal.pone.0328047)
Supplement: S5 Table — (DOCX) [file pone.0328047.s006.docx]

**Table 5. Value ranges for each parameter**

| **Parameter** | **Value** |
| --- | --- |
| **Tube thickness(*t*_s_)** | 2-11(mm) |
| **diameter-to-thickness ratio(*D*/*t*_s_)** | 20-57 |
| **yield strength of steel tubes(*f*_ys_)** | 205-460(MPa) |
| **number of CFRP layers(*n*_c_)** | 4-10 |
| **tensile strength of CFRP(*f*_ct_)** | 2500-4000(MPa) |
