# Supplementary material for: Static strengths of circular hollow section stub column strengthened with carbon fiber reinforced polymer
Source: PLoS One. 2025 Aug 1;20(8):e0328047. doi: 10.1371/journal.pone.0328047 (PMC12316273; doi:10.1371/journal.pone.0328047)
Supplement: S6 Table — (DOCX) [file pone.0328047.s007.docx]

**Table 6. Geometric details of CHS stub columns**

| **No.** | ***D*/mm** | ***t*_s_/mm** | ***L*_s_/mm** | ***D*/*t*_s_** |
| --- | --- | --- | --- | --- |
| **1** | 102.0 | 2.0 | 350.0 | 51.0 |
| **2** | 114.0 | 2.0 | 400.0 | 57.0 |
| **3** | 102.0 | 3.0 | 350.0 | 34.0 |
| **4** | 114.0 | 3.0 | 400.0 | 38.0 |
| **5** | 102.0 | 4.0 | 350.0 | 25.5 |
| **6** | 114.0 | 4.0 | 400.0 | 28.5 |
| **7** | 168.0 | 4.0 | 600.0 | 42.0 |
| **8** | 203.0 | 4.0 | 700.0 | 50.8 |
| **9** | 102.0 | 5.0 | 350.0 | 20.4 |
| **10** | 114.0 | 5.0 | 400.0 | 22.8 |
| **11** | 168.0 | 5.0 | 600.0 | 33.6 |
| **12** | 325.0 | 8.0 | 900.0 | 40.6 |
| **13** | 406.0 | 8.0 | 1500.0 | 50.8 |
| **14** | 406.0 | 9.0 | 1500.0 | 45.1 |
| **15** | 406.0 | 10.0 | 1500.0 | 40.6 |
| **16** | 406.0 | 11.0 | 1500.0 | 36.9 |

Note: *D*, *t*_s_ and *L*_s_ represent the outer diameter, the tube thickness and the length of steel tube respectively.
