# Supplementary material for: Static strengths of circular hollow section stub column strengthened with carbon fiber reinforced polymer
Source: PLoS One. 2025 Aug 1;20(8):e0328047. doi: 10.1371/journal.pone.0328047 (PMC12316273; doi:10.1371/journal.pone.0328047)
Supplement: S7 Table — (DOCX) [file pone.0328047.s008.docx]

**Table 7. The confinement coefficient *λ* at different numbers of CFRP layers**

| **Yield strength**  **(MPa)** | **confinement coefficient *λ*** | | | |
| --- | --- | --- | --- | --- |
|  | **1 layer** | **2 layers** | **3 layers** | **4 layers** |
| **245** | 0.41 | 0.82 | 1.21 | 1.64 |
| **335** | 0.30 | 0.61 | 0.90 | 1.20 |
| **345** | 0.29 | 0.58 | 0.87 | 1.16 |
| **420** | 0.24 | 0.48 | 0.71 | 0.95 |
| **460** | 0.22 | 0.44 | 0.65 | 0.87 |
