# Supplementary material for: Static strengths of circular hollow section stub column strengthened with carbon fiber reinforced polymer
Source: PLoS One. 2025 Aug 1;20(8):e0328047. doi: 10.1371/journal.pone.0328047 (PMC12316273; doi:10.1371/journal.pone.0328047)
Supplement: S8 Table — (DOCX) [file pone.0328047.s009.docx]

**Table 8. Strengthening schemes**

| **Group** | **Configuration** | |
| --- | --- | --- |
|  | **Hoop** | **Hoop and longitudinal** |
| **1** | 2H | 1H1L |
| **2** | 4H | 2H2L |
| **3** | 6H | 3H3L |
