# Supplementary material for: Static strengths of circular hollow section stub column strengthened with carbon fiber reinforced polymer
Source: PLoS One. 2025 Aug 1;20(8):e0328047. doi: 10.1371/journal.pone.0328047 (PMC12316273; doi:10.1371/journal.pone.0328047)
Supplement: S9 Table — (DOCX) [file pone.0328047.s010.docx]

**Table 9.** **Ultimate strength of CHS stub columns after CFRP strengthening**

| **Group** | **Configuration** | **Ultimate strength**  **kN** |
| --- | --- | --- |
| **1** | 2H | 481.2 |
|  | 1H1L | 462.6 |
| **2** | 4H | 541.5 |
|  | 2H2L | 482.2 |
| **3** | 6H | 581.7 |
|  | 3H3L | 502.2 |
